# Human histone pre-mRNA assembles histone or canonical mRNA-processing complexes by overlapping 3′-end sequence elements

**DOI:** 10.1093/nar/gkac878

**Published:** 2022-11-30

**Authors:** Francesco S Ielasi, Sara Ternifi, Emeline Fontaine, Domenico Iuso, Yohann Couté, Andrés Palencia

**Affiliations:** Institute for Advanced Biosciences (IAB), Structural Biology of Novel Targets in Human Diseases, INSERM U1209, CNRS UMR5309, Université Grenoble Alpes, Grenoble, France; Institute for Advanced Biosciences (IAB), Structural Biology of Novel Targets in Human Diseases, INSERM U1209, CNRS UMR5309, Université Grenoble Alpes, Grenoble, France; Institute for Advanced Biosciences (IAB), Structural Biology of Novel Targets in Human Diseases, INSERM U1209, CNRS UMR5309, Université Grenoble Alpes, Grenoble, France; Institute for Advanced Biosciences (IAB), Epigenetics and Cell Signaling, INSERM U1209, CNRS UMR5309, Université Grenoble Alpes, Grenoble, France; Université Grenoble Alpes, INSERM, CEA, UMR BioSanté U1292, CNRS, CEA, FR2048, 38000 Grenoble, France; Institute for Advanced Biosciences (IAB), Structural Biology of Novel Targets in Human Diseases, INSERM U1209, CNRS UMR5309, Université Grenoble Alpes, Grenoble, France

## Abstract

Human pre-mRNA processing relies on multi-subunit macromolecular complexes, which recognize specific RNA sequence elements essential for assembly and activity. Canonical pre-mRNA processing proceeds via the recognition of a polyadenylation signal (PAS) and a downstream sequence element (DSE), and produces polyadenylated mature mRNAs, while replication-dependent (RD) histone pre-mRNA processing requires association with a stem–loop (SL) motif and a histone downstream element (HDE), and produces cleaved but non-polyadenylated mature mRNAs. H2AC18 mRNA, a specific H2A RD histone pre-mRNA, can be processed to give either a non-polyadenylated mRNA, ending at the histone SL, or a polyadenylated mRNA. Here, we reveal how H2AC18 captures the two human pre-mRNA processing complexes in a mutually exclusive mode by overlapping a canonical PAS (AAUAAA) sequence element with a HDE. Disruption of the PAS sequence on H2AC18 pre-mRNA prevents recruitment of the canonical complex *in vitro*, without affecting the histone machinery. This shows how the relative position of *cis*-acting elements in histone pre-mRNAs allows the selective recruitment of distinct human pre-mRNA complexes, thereby expanding the capability to regulate 3′ processing and polyadenylation.

## INTRODUCTION

Processing of 3′-untranslated regions (UTRs) of precursor mRNAs (pre-mRNA) is a fundamental step in mRNA maturation, during which the mRNA transcript is cleaved at a specific site, located downstream of the open reading frame of the encoded gene. The position of the cleavage site, generally occurring after a CA dinucleotide, is determined by *cis-*acting RNA sequence elements, which are recognized by large processing complexes (1–3).

Two main mechanisms of metazoan 3′-pre-mRNA processing have been identified so far, involving either canonical mRNAs during all phases of the cell cycle (4,5) or replication-dependent (RD) histone mRNAs mainly during the S phase (6,7). The vast majority of mRNA transcripts undergo canonical processing, during which a polyadenylation signal (PAS) sequence element, located ∼20 nucleotides upstream of the cleavage site, is recognized by the cleavage and polyadenylation specificity factor (CPSF) complex, specifically by the WDR33 and CPSF30/CPSF4 subunits, assembled by the CPSF160/CPSF1 scaffold in the mammalian polyadenylation specificity factor (mPSF) module (8–13). Other CPSF-associated subcomplexes further ensure correct RNA positioning for processing, specifically the cleavage stimulation factor (CstF) —recognizing a U/G-rich sequence downstream of the cleavage site and essential for pre-mRNA processing— and the cleavage factor Im (CF Im) that recognizes UGUA motifs upstream of the cleavage site and stimulates CPSF activity, though is not strictly required for the latter (14,15). The subsequent cleavage reaction is catalyzed by the endonuclease CPSF73, followed by the addition of a polyadenosine tail by poly(A) polymerase (PAP), associated with the mPSF subcomplex via the scaffold Fip1 (9,16).

CPSF73 is part of the mammalian cleavage factor (mCF) module also containing the scaffold protein Symplekin (SYMPK) and CPSF100/CPSF2. These three proteins form a complex which is highly dynamic in its inactive state (13) and exhibits low specificity and activity as a stand-alone endonuclease module (17). Notably, the CPSPF73/CPSF100/SYMPK endonuclease complex, when bound to CstF64/CSTF2, is also recruited to the histone mRNA processing complex (18–21), named the histone cleavage complex (HCC). A recent structure of recombinant human HCC was solved in complex with mouse histone H2A pre-mRNA and U7 small nuclear ribonucleoprotein (snRNP) (22); it represents the first observation of the cleavage module in an active, mRNA-bound state. In the core of the structure, the histone downstream element (HDE) present in mouse H2A.2 pre-mRNA base-pairs with U7 small nuclear RNA (U7 snRNA) within the heptameric U7 snRNP, including five Sm (SmB/D3/E/F/G) and two Lsm (Lsm10/11) subunits. The formation of the HDE–U7 duplex with the pre-assembled U7 snRNP/HCC/FLASH (FLASH=FLICE-associated huge protein) complex in the histone locus bodies (HLBs) during the S phase, and its interaction with the N-terminal domain of SYMPK and CPSF100, have been proposed to trigger structural rearrangements within the HCC module needed for the activation of mRNA processing by CPSF73 (19,22–24). After pre-mRNA cleavage, CPSF73 acts as a 5′–3′ exonuclease and completes the histone pre-mRNA maturation cycle by degrading the 3′ fragment of the cleaved mRNA (25). Despite its association with SYMPK as a component of the HCC, CstF64 was not observed in single-particle electron microscopy (EM) analysis, and its presence was found to be non-essential for histone mRNA cleavage (22) *in vitro*.

Canonical pre-mRNA polyadenylation is essential to ensure nuclear export, cytoplasmic stability and translation of RNA transcripts. In the processing of RD histone pre-mRNAs, which are not polyadenylated, an analogous role is played by the interaction of a stem–loop (SL) motif, located upstream of the endonuclease cleavage site, with the stem–loop-binding protein (SLBP) (26–28). This interaction promotes RD histone pre-mRNA association with U7 snRNP and FLASH (24), and its disruption causes degradation of histone mRNAs (29). Exceptions to this rule are replication-independent histone variants, which are polyadenylated (30), and histone H2A.X, involved in DNA damage repair, which can undergo canonical or histone processing depending on the cell type (31,32). Furthermore, polyadenylated transcripts of a subset of RD histone genes —including H1C (HIST1H1C) and H2AC18 (HIST2H2AA3)— have been found in human and other mammalian tissues. In particular, the mouse ortholog of HIST2H2AA3 was shown to be expressed as a polyadenylated transcript in differentiated fibroblasts, which could be a mechanism to compensate for the loss of histone copies in non-dividing tissues (33). Interestingly, H1C and H2AC18 carry a PAS motif in an unusual position, which precedes and partially overlaps the HDE region. However, the molecular mechanisms controlling the switch between classical histone mRNA processing and polyadenylation of these RD histone mRNAs are unknown.

In this work, we show that the 3′-UTR of H2AC18 pre-mRNA, which contains an HDE sequence and a PAS (AAUAAA) motif, can efficiently capture not only the histone 3′-pre-mRNA processing complex but also the canonical pre-mRNA processing complexes. Interestingly, H2AC18 pre-mRNA contains an uncommon 3′-end pre-mRNA architecture compared with classical RD histone transcripts, due to the overlap of HDE and PAS sequence elements. We show that by intercalating the PAS within the HDE, H2AC18 pre-mRNA deploys a novel mechanism to recruit the canonical or the histone pre-mRNA-processing complexes in a mutually exclusive manner. We validate this finding either by using an antisense U7 (aU7) oligonucleotide or by disrupting the PAS site, thus preventing the assembly of the histone or the canonical pre-mRNA-processing complexes, respectively. Our work shows how human RD pre-mRNAs can control their 3′-end processing by strategic arrangement of mRNA *cis*-acting elements to selectively recruit distinct 3′-pre-mRNA-processing complexes.

## MATERIALS AND METHODS

### Cell culture conditions

All human cell lines used in this work were grown at 37°C in a moist atmosphere containing 5% CO_2_. Human embryonic kidney-derived freestyle cells (HEK293-F cells, RRID: CVCL_D603) were a gift from Delphine Guilligay (IBS, Grenoble, France). Henrietta Lacks patient-derived-S3 cells (HeLa S3, RRID: CVCL_0058) were a gift from Dr Nicolas Reynoird (IAB, La Tronche, France). Suspension HEK293-F cells were cultured in suspension either in Ex-Cell medium (Sigma), supplemented with l-glutamine, or in FreeStyle medium (ThermoFisher), and harvested at a cell density of ∼2.5–3 × 10^6^ cells/ml. Adherent human HeLa S3 cells were grown in T-75 flasks in Dulbecco's modified Eagle’s medium (DMEM) supplemented with 10% fetal bovine serum (FBS). When required for nuclear extraction, HeLa cells were trypsinized and transferred in Erlenmeyer flasks for growth in suspension. In this case, cells were placed under continuous stirring and harvested at a cell density of ∼1 × 10^6^ cells/ml. Cells were not re-authenticated by the laboratory.

Adipose-derived mesenchymal stem cells (MSCs; ATCC PCS-500-011) were grown in T-75 or T150 flasks in mesenchymal stem cell growth medium 2 (PromoCell). At passage 8, cells were either trypsinized for nuclear extract preparation or used for differentiation into adipocytes. Differentiation was induced with the MesenCult Adipogenic Differentiation Kit (StemCell Technologies), by refreshing differentiation medium every 3 days. Adipocytes were harvested for nuclear extract preparation after 12 days of differentiation, characterized by the extensive appearance of intracellular lipid droplets. Nonetheless, differentiation was verified by conventional staining with Oil Red.

### Antibodies

CPSF30 (A301-585A), CPSF73 (A301-091A), CPSF160 (A301-580A), SYMPK (A301-465A), SLBP (A303-968A), CstF-64 (A301-092A) and SmD3 (A303-954A) rabbit primary antibodies were purchased from Bethyl Laboratories. Lsm11(26119-1-AP) and Nucleolin (10556-1-AP) rabbit primary antibodies were purchased from Proteintech. The secondary horseradish peroxidase (HRP) AffiniPure goat anti-rabbit antibody (111-035-144) was manufactured by Jackson ImmunoResearch.

### RNA oligonucleotides

All RNA oligonucleotides were synthesized by IDT. A comprehensive list of RNA oligonucleotides used in this study and an exhaustive description of their structures and functions are reported in Supplementary Table S1. The photocleavable biotin moiety is indicated in the sequences as ‘PCBiotin’, the 18-atom spacer as ‘18SP’ and 2′*-O*-methylated nucleotides spanning the CA cleavage site (for the inhibition of RNA cleavage) with an ‘m’.

### Preparation of nuclear extracts from cell cultures

Cells were recovered either by centrifugation, in the case of HEK/HeLa suspension cultures (1000 *g*, 15 min, 22°C), or trypsinized, in the case of adherent human MSCs (hMSCs) or adipocytes, then washed three times with ice-cold phosphate-buffered saline (PBS) and resuspended for 10 min in 3–5 times the cell pellet volume of buffer A [10 mM HEPES-KOH, pH 8.0, 10 mM KCl, 1.5 mM MgCl_2_, 0.5 mM dithiothreitol (DTT), 0.75 mM spermidine, 0.15 mM spermine] to allow swelling. Cells were then lysed using a Dounce homogenizer (10 strokes), and lysis was checked under the microscope by staining with trypan blue. A volume of buffer B (50 mM HEPES-KOH, pH 8.0, 67% sucrose, 0.75 mM spermidine, 0.15 mM spermine, 10 mM KCl, 0.2 mM EDTA, 0.1 mM DTT) corresponding to 10% of the cell lysate volume was added. Nuclei were recovered by centrifugation (1000 *g*, 10 min, 4°C). For HEK/HeLa nuclei, after estimation of the packed nuclei volume, a volume of buffer C [20 mM HEPES-KOH, pH 8.0, 25% glycerol, 1.5 mM MgCl_2_, 0.6 M KCl, 0.2 mM EDTA, 0.5 mM DTT, 0.5 mM phenylmethyl sulfonylfluoride (PMSF)] was added in order to reach a KCl concentration of ∼0.24–0.25. For hMSC/adipocytes, which yielded much smaller nuclear pellets, nuclei were resuspended with buffer A to a total volume of ∼600 μl, to which an appropriate volume of buffer C was added (∼400 μl) in order to reach a KCl concentration of 0.24 M. In all cases, after homogenization using a Dounce pestle, the mixture was placed under gentle stirring for 1 h at 4°C, then centrifuged (10 000 *g*, 30 min, 4°C). The recovered supernatant was transferred to a 10 kDa dialysis cassette and dialyzed overnight or for 4 h against a minimum of 100 times the supernatant volume of buffer D (20 mM HEPES-KOH, pH 8.0, 20% glycerol, 0.1 M KCl, 0.2 mM EDTA, 0.5 mM DTT, 0.5 mM PMSF). The nuclear extract was then centrifuged to remove precipitated material (10 000 *g*, 20 min, 4°C), flash-frozen in liquid nitrogen and stored at –80°C.

### Preparation of nuclear extracts from mouse liver tissues

All mouse experimental protocols were approved by the official ethics committee of the University Grenoble Alpes (ComEth, C2EA-12). Mice were bred in the animal facility (Grenoble High Technology Animal Facility - PHTA, University Grenoble Alpes). Female mice of 2–5 months of age were used for this study. C57BL/6N mice were euthanized, and the livers were collected immediately. Livers (from five mice) were homogenized in cold buffer (3 ml/each liver) containing 2.2 M sucrose, 10 mM Tris–HCl pH 7.5, 10 mM MgCl_2_ and protease inhibitor (Roche), filtered by hydrophilic gauze and centrifuged for 3 h at 100 000 *g* at 4°C. Nuclei in the pellet were washed in a buffer containing 0.5 M sucrose, 10 mM Tris–HCl pH 7.5, 10 mM MgCl_2_, 0.2% Triton X-100 and protease inhibitor, centrifuged 20 min at 1500 *g* at 4°C and resuspended in 0.5 ml of buffer containing 20 mM HEPES-KOH, pH 8.0, 25% glycerol, 1.5 mM MgCl_2_, 0.25 M KCl, 0.2 mM EDTA, 0.5 mM DTT, 0.5 mM PMSF. After homogenization using a Dounce pestle, the mixture was placed under gentle stirring for 1 h at 4°C, then centrifuged (10 000 *g*, 20 min, 4°C). The recovered supernatant was transferred to a 10 kDa dialysis cassette and dialyzed for 4 h against 20 mM HEPES-KOH, pH 8.0, 20% glycerol, 0.1 M KCl, 0.2 mM EDTA, 0.5 mM DTT, 0.5 mM PMSF. The nuclear extract was then centrifuged to remove precipitated material (10 000 *g*, 15 min, 4°C), flash-frozen in liquid nitrogen and stored at –80°C.

### Complex assembly and purification

All stock solutions of the synthetic RNA sequences used here were annealed in binding buffer (15 mM HEPES-KOH, pH 8.0, 15% glycerol, 75 mM KCl, 10 mM EDTA) by incubation at 90°C for 5 min and cooling on ice at 5°C. To each nuclear extract sample, 80 mM EDTA pH 8 was added to reach a final EDTA concentration of 20 mM. For negative control samples, SL RNA and aU7 snRNA were pre-incubated with nuclear extracts, at a concentration of 10 μg/ml and 20 μg/ml, respectively (unless otherwise specified), for 20 min on ice. For samples containing N-FLASH, nuclear extracts containing the recombinant protein at a concentration of 50 μg/ml were incubated on ice for 30 min before addition of H2A_4m. The RNA oligonucleotides H4_1m or H2A_4m were then added to nuclear extracts to a concentration of 40 nM, unless otherwise specified. In the case of experiments with synthetic U7 snRNA, a pre-annealed equimolar mixture of H2A_4m and U7 was used and added to nuclear extracts to a concentration of 40 nM. The RNA–nuclear extract mixtures were next incubated at 12°C for 10 min, cooled down on ice and centrifuged to remove precipitated material (10 000 *g*, 10 min, 4°C). Streptavidin–agarose beads (Merck), previously equilibrated with binding buffer, were added and the suspensions were placed on a rotating wheel for 90 min at 4°C, in order to allow binding of the complexes onto the beads. These were subsequently recovered by low-speed centrifugation (20 *g*, 3 min, 4°C), washed three times with binding buffer and resuspended in 2–3 bead volumes of the same buffer. Bead suspensions were then transferred to new 0.5 ml tubes, placed on a Petri dish filled with ice and the RNA–protein complexes were eluted by irradiation for a total of 30 min with a 100 W UV lamp (Cole-Parmer) at 366 nm. After every 5 min of UV exposure, the tubes were vortexed and placed on a new ice bed to ensure uniform exposure and prevent overheating. Eluted pre-mRNA processing complexes were finally recovered by centrifugation (20 *g*, 3 min, 4°C) and supernatant collection, and either used for sodium dodecylsulfate (SDS)–polyacrylamide gel electrophoresis (PAGE) and subsequent western blot or mass spectrometry (MS) analysis, or flash-frozen in liquid nitrogen and stored at –80°C for later use.

### Protein cross-linking, electrophoresis and western blotting

SDS–PAGE was performed on 4–15% TGX Stain Free Gels (Biorad). Native PAGE was performed using the Novex NativePAGE system, including Bis-Tris NativePAGE 3–12% gels (Thermo Fisher Scientific). Before native PAGE experiments, samples were cross-linked with the DTSSP cross-linker, added to a final concentration of 1 mM, and incubated in ice for 1 hr. The cross-linking reaction was quenched by addition of Tris–HCl buffer pH 7.5 to a final concentration of 20 mM and incubation in ice for 15 min.

Western blotting was performed by electrotransfer from SDS–PAGE or native PAGE gels onto polyvinylidene difluoride (PVDF) membranes (GE Healthcare) for 30 min at 25 V. Membranes were blocked with 5% milk powder in PBS buffer containing 0.05% Tween-20, then incubated with primary antibodies diluted in PBS buffer containing 1% bovine serum albumin (BSA) and 0.05% Tween-20, either for 3 h at room temperature or overnight at 4°C. Membranes were washed three times with 0.05% Tween-20 in PBS buffer, then incubated with secondary antibody in BSA–Tween–PBS buffer at room temperature for 1 hr. After three washes, membranes were developed by incubation with the ECL RevelBlot Plus reagents (Ozyme) and subsequent acquisition with the Fusion FX system (Vilber).

### Histone mRNA cleavage activity assay

Nuclear extracts prepared from suspension cultures of human HEK293-F cells were used in 3′ histone pre-mRNA cleavage assays. At the beginning of each experiment, 80 mM EDTA pH 8 was added to each nuclear extract sample to a final concentration of 20 mM. For negative control samples, SL RNA and aU7 snRNA were pre-incubated with nuclear extracts, at a concentration of 10 μg/ml and 20 μg/ml, respectively, for 20 min on ice. The wild-type H2AC18 oligonucleotide used here was annealed in binding buffer (15 mM HEPES-KOH, pH 8.0, 15% glycerol, 75 mM KCl, 10 mM EDTA), by incubation at 90°C for 5 min and cooling on ice at 5°C.

In cleavage assays performed with crude nuclear extracts, wild-type H2AC18 oligonucleotide was added to nuclear extracts to a concentration of 1.6 nM and the mixtures were incubated for 10 min on ice, to allow histone mRNA processing complex assembly. Endonucleolytic cleavage of wild-type H2AC18 was performed by incubating samples for 2 h at 32°C with gentle mixing. The reaction was stopped by heating for 5 min at 92°C in the presence of 0.3 M sodium acetate, and RNA was isolated by phenol–chloroform extraction and ethanol precipitation overnight at –20°C.

In assays performed with purified streptavidin-bound complexes, wild-type H2AC18 RNA was added to nuclear extracts to a concentration of 3.5 nM and, after incubation for 10 min on ice, the mixtures were centrifuged to remove precipitated material (10 000 *g*, 10 min, 4°C) and applied onto streptavidin–agarose beads. The resulting suspensions were placed on a rotating wheel for 90 min at 4°C, in order to allow binding of the complexes onto the beads. These were subsequently recovered by low speed centrifugation (20 *g*, 3 min, 4°C), washed twice with binding buffer and resuspended in 4 bead volumes of the same buffer. Endonucleolytic cleavage of wild-type H2AC18 RNA was performed by incubating samples at 32°C with gentle mixing for 2 hr. The reaction was stopped by heating for 5 min at 92°C in the presence of 2% SDS, and RNA was extracted by using the NucleoSpin miRNA kit (Macherey-Nagel) and stored at –80°C before use.

Northern blot RNA analysis was performed following the procedure described by Rio (34). Extracted RNA samples were resuspended in formamide-containing RNA gel loading dye (Thermo Fisher Scientific), loaded onto 10% TBE–urea PAGE gels (BioRad) pre-run for 30 min at 80 V, and run at 150 V for 1 hr. Electrotransfer was performed on Hybond-N+ membranes (GE Healthcare) in ice for 2 h at 250 mA and for another 2 h at 300 mA. After transfer, RNA was cross-linked to membranes by UV irradiation. The biotinylated RNA cleavage substrate and products were revealed with the Chemiluminescent Nucleic Acid Detection Module Kit (Thermo Fisher Scientific) and subsequent membrane luminescence acquisition with the Fusion FX system (Vilber).

### Expression and purification of N-FLASH

The N-terminus of human FLASH/CASP8AP2 (N-FLASH, residues 51–138, Uniprot ID: Q9UKL3) was codon optimized for expression in bacteria, synthesized and inserted within pET26-b(+) vector by Genscript, using NdeI and BamHI sites, resulting in an N-terminally 6×His tagged construct. The recombinant protein was overexpressed in BL21(DE3)-CodonPlus-RIL bacteria and purified by a procedure adapted from Aik *et al.* (35). Briefly, bacterial cultures were induced with 0.5 mM isopropyl-β-d-thiogalactopyranoside and grown for 16 h at 18°C. After centrifugation at 4000 *g* for 20 min at 4°C, bacterial pellets were resuspended in lysis buffer containing 20 mM Tris–HCl pH 7.5, 500 mM NaCl, 5% glycerol, 10 mM imidazole, 10 mM β-mercaptoethanol and protease inhibitor cocktail (Roche), and lysed by sonication on ice. Bacterial lysates were centrifuged at 15 000 *g* for 30 min at 4°C, then applied to pre-equilibrated Ni-NTA resin by gravity flow. The resin was extensively washed with lysis buffer and protein was eluted with buffer containing 20 mM Tris–HCl pH 7.5, 500 mM NaCl, 400 mM imidazole, 10 mM β-mercaptoethanol and protease inhibitors. A further purification and buffer exchange step by size exclusion chromatography was performed on a Superdex 200 column (Cytiva), pre-equilibrated with a buffer compatible with histone 3′-mRNA-processing complex purification and containing 250 mM KCl, 20 mM HEPES-KOH and 5 mM DTT. Peak fractions containing N-FLASH were pooled, concentrated to 5 mg/ml, flash-frozen in liquid nitrogen and stored at –80°C.

### Mass spectrometry-based quantitative proteomics

Three biological replicates of complex purification using different RNA baits (H2A_4m and H2A_4m + U7/SL RNAs for the first experiment, and H2A_4m RNA, H2A_4m + aU7/SL RNAs and 38G/40A H2A_4m RNA for the second experiment) were performed from nuclear extracts of HEK293-F cells. Three biological replicates of the complex purification using H2A_4m RNA were performed from nuclear extracts of hMSCs and adipocytes. Finally, complex purifications using H2A_4m and 38G/40A H2A_4m RNAs were performed from the nuclear extract of mouse livers. The eluted proteins were solubilized in Laemmli buffer and stacked on the top of a 4–12% NuPAGE gel (Thermo Fisher Scientific). After staining with R-250 Coomassie Blue (Biorad), proteins were digested in-gel using trypsin (modified, sequencing purity, Promega), as previously described (36).

The resulting peptides were analyzed by online nanoliquid chromatography coupled to MS/MS (Ultimate 3000 RSLCnano and Q-Exactive HF or Q-Exactive Plus, Thermo Fisher Scientific for human and mouse samples, respectively) using a 120 or 80 min gradient for human and mouse samples, respectively. For this purpose, the peptides were sampled on a pre-column (300 μm × 5 mm PepMap C18, Thermo Fisher Scientific) and separated in a 75 μm × 250 mm C18 column (Reprosil-Pur 120 C18-AQ, 1.9 μm, Dr Maisch). The MS and MS/MS data were acquired by Xcalibur (Thermo Fisher Scientific).

Peptides and proteins were identified by Mascot (version 2.7.0 and version 2.8.0, Matrix Science, for, respectively, HEK293-F samples and hMSC, adipocyte and mouse liver samples) through concomitant searches against the Uniprot database (*Homo sapiens* taxonomy, May 2021 download for HEK293-F samples and May 2022 download for hMSC and adipocyte samples, or *Mus musculus* taxonomy, March 2022 download for mouse samples), a homemade database containing the sequences of classical contaminant proteins found in proteomic analyses (human keratins, trypsin, etc.), and the corresponding reversed databases. Trypsin/P was chosen as the enzyme, and two missed cleavages were allowed. Precursor and fragment mass error tolerances were set respectively at 10 and 20 ppm. Peptide modifications allowed during the search were: carbamidomethyl (C, fixed), acetyl (Protein N-term, variable) and oxidation (M, variable). The Proline software (37) was used for the compilation, grouping and filtering of the results [conservation of rank 1 peptides, peptide length ≥6 amino acids, peptide score ≥25, allowing to reach a false discovery rate (FDR) of peptide–spectrum match identifications <1% as calculated on peptide–spectrum match scores by employing the reverse database strategy, and a minimum of one specific peptide per identified protein group]. Proline was then used to perform a spectral counting-based comparison or a MS1 label-free quantification based on razor and specific peptides of the identified protein groups.

MS data have been deposited at the ProteomeXchange Consortium via the PRIDE partner repository (35) with the dataset identifiers PXD027636 and PXD034984, and are publicly available as of the date of the publication.

Statistical analysis was performed using the ProStaR software (38) on the basis of the quantitative data obtained with the three biological replicates analyzed per condition. Proteins identified in the contaminant database, proteins identified by MS/MS in fewer than two replicates of one condition and proteins detected in fewer than three replicates of one condition were discarded. After log_2_ transformation, abundance values were normalized by median centering, before missing value imputation (slsa algorithm for partially observed values in the condition and DetQuantile algorithm for totally absent values in the condition). Statistical testing was conducted with limma, whereby differentially expressed proteins were sorted out using a log_2_(fold change) cut-off of 1 and a *P*-value cut-off of 0.01, leading to an FDR <1.3% according to the Benjamini–Hochberg estimator.

Intensity-based absolute quantification (iBAQ) (39) values were calculated from MS1 intensities of razor and specific peptides. The iBAQ values were normalized by the sum of iBAQ values in the sample, before summing the values of the three replicates to generate the final iBAQ value of each condition.

### Structural modeling and representation

The 3D model of the histone 3′-pre-mRNA-processing machinery, in complex with the 3′-UTR of the human wild-type H2AC18 pre-mRNA, was built by using as a template the cryo-EM structure of the machinery core bound to the mouse H2A pre-mRNA [Protein Data Bank (PDB) accession code: 6V4X] (22). The H2AC18 pre-mRNA was modeled with the Coot software (40), by changing the nucleotides of mouse H2A pre-mRNA into those of human H2AC18 pre-mRNA, followed by manual adjustment and execution of local energy refinements. Structural figures were prepared using the ChimeraX molecular graphics software (41)

## RESULTS

### Human histone H2AC18 and H4 pre-mRNAs associate with the shared 3′-cleavage factor module and support 3′ processing

**Figure 1.**
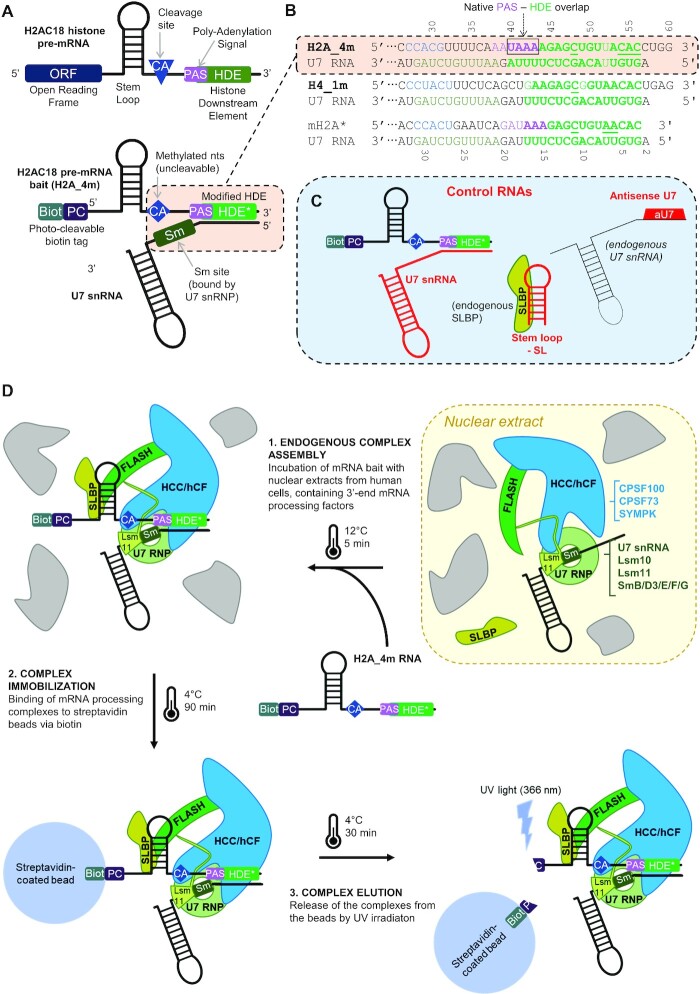
Strategy for purification of endogenous human 3′-pre-mRNA-processing complexes. (**A**) RNA sequences were designed based on the 3′-UTR of histone pre-mRNAs (upper structure), with an SL motif and the HDE sequence, mediating the interaction with SLBP and the U7 snRNA (lower structure), respectively; the cleavage site (blue diamond) was modified to avoid mRNA cleavage during complex isolation; a 5′-biotin and an adjacent photocleavable tag allow for complex immobilization on streptavidin beads and subsequent elution by UV irradiation. (**B**) Alignment of the pre-mRNA bait sequences with the endogenous human U7 snRNA sequence; the region between the modified CA cleavage site and the duplex-forming HDE is shown: 2′-*O*-Methylated nucleotides spanning the cleavage site are indicated in cyan, the pairing bases are indicated in bold green, and the residues modified in the HDE to improve complementarity with U7 RNA are underlined; in H2A_4m, the PAS region, superimposed on the HDE, is indicated in violet; in U7 RNA, the Sm-interacting region is shown in dark green. (**C**) Schemes of the RNA molecules used to selectively control the assembly of the histone 3′-mRNA-processing complex on the bait pre-mRNAs. (**D**) mRNA-processing complex purification workflow. Step 1: incubation of nuclear extracts, containing 3′-end pre-mRNA cleavage factors, with pre-mRNAs. Step 2: addition of streptavidin-coated beads to the mixture, allowing specific immobilization of RNA bait and associated protein complexes via the biotin moiety of pre-mRNA bait. Step 3: elution of RNA and associated protein complexes by UV irradiation. The schematic shows as example the purification of the histone pre-mRNA-processing complex associated with an H2A_4m molecule; simultaneous assembly and purification of the canonical pre-mRNA-processing machinery (also present in nuclear extracts), bound to the PAS region of a separate mRNA strand, follows the same workflow.

In order to capture the human histone 3′-pre-mRNA complex and assess its endonuclease activity, we designed RNA baits from human H2AC18 (HIST2H2AA3) and H4C11 (HIST1H4J) pre-mRNAs. These sequences contain a 5′-photocleavable biotin tag to allow purification on streptavidin beads (Figure 1A, B; Supplementary Table S1), a strategy successfully used for *Drosophila* and mouse complexes in the past (20,23). We designed 60 nt long pre-mRNAs of H2AC18 and H4C11 (herein H2A_4m and H4_1m, respectively), including the SL and HDE regions, and the cleavage sites between them. To increase complementarity with the endogenous U7 snRNA sequence, the HDE sequences were slightly modified (HDE*, 4 nt in H2A_4m and 1 nt in H4_1m, underlined residues in Figure 1B). The same strategy was applied for the mouse sequence mH2a* (20,22), which facilitated the purification of the associated complex without significantly perturbing its activity or stability. 2′-*O*-Methylated nucleotides spanning the CA cleavage site were introduced to prevent RNA cleavage (42) and subsequent complex release (Figure 1A; Supplementary Table S1).

Interestingly, the sequence of human H2AC18 pre-mRNA slightly differs from that of its mouse ortholog, as it contains a common AAUAAA PAS sequence (spanning residues 38–43). However, both human AAUAAA and mouse GAUAAA sequences are functional PAS sequences (33,43) located in an unusual position, where the PAS is not only placed before the HDE but also overlapping the 5′ region of the latter (Figure 1A, B; Supplementary Figure S1A–D; Supplementary Table S1). Conversely, human H4 pre-mRNA has a completely different architecture as it contains a PAS sequence upstream of the SL motif (Figure 1B; Supplementary Figure S1A–D; Supplementary Table S1).

As controls to study complex assembly, we used: (i) U7 snRNA pre-annealed with our RNA baits before purification of complexes; (ii) an aU7 added to nuclear extracts to block the endogenous U7 snRNA and impair duplex formation with the RNA baits; and (iii) an SL RNA to deplete endogenous SLBP (Figure 1C; Supplementary Table S1).

**Figure 2.**
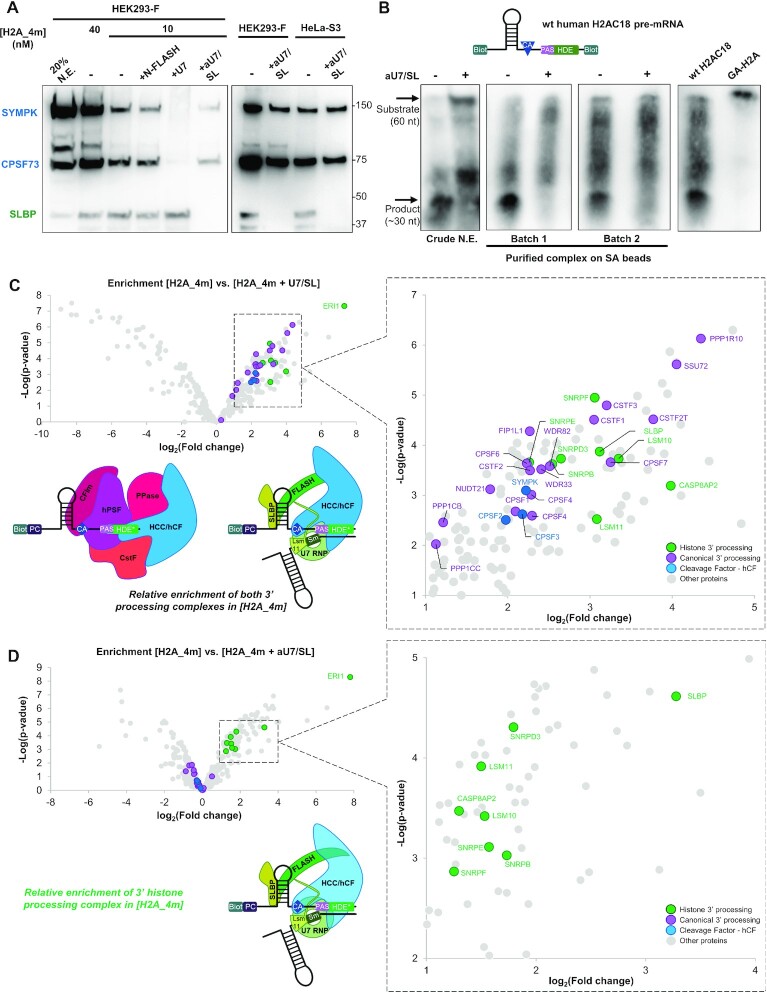
The 3′-UTR of histone H2AC18 pre-mRNA can assemble both histone and canonical 3′-mRNA-processing complexes. (**A**) Western blot analysis of complexes purified from HEK293-F nuclear extract (N.E.) with the H2A_4m RNA, prepared with different oligonucleotide concentrations, in the absence or presence of different control molecules (U7, aU7 + SL) or in the presence of the recombinant N-terminal FLASH (N-FLASH) (left subpanel); comparison of results obtained with HEK293-F and HeLa-S3, both using 40 nM H2A_4m (right subpanel). (**B**) Activity assay with a double-biotin wild-type (wt) H2AC18 3′-UTR sequence (structure on top), performed either in crude HEK293-F nuclear extract or with purified histone RNA-processing complex; cleavage products were purified and analyzed by northern blot and luminescent biotinylation detection; complex assembly and cleavage were blocked in the presence of aU7/SL oligonucleotides; the uncleavable GA-H2A was used as weight standard. (**C**, **D**) Differential analyses of protein abundances measured by MS-based quantitative proteomics: [H2A_4m] *versus* [H2A_4m + U7/SL] samples (C); [H2A_4m] *versus* [H2A_4m + aU7/SL] (D). The volcano plots represent the –log_10_(limma *P*-value) on the *y*-axis plotted against the log2(fold change) on the *x*-axis. Relevant identified proteins that are at least 2-fold more abundant with [H2A_4m] compared with [H2A_4m + U7/SL] or [H2A_4m + aU7/SL] are annotated in the zoom-in graphs (subpanels on the left); schematic representations of complex relative abundances are shown (lower subpanels). Abbreviations: CFIm, ceavage factor 1m module (including NUDT21, CPSF6 ands CPSF7 subunits); CPSF, cleavage and polyadenylation specificity factor; CstF, cleavage-stimulating factor module (including CSTF1, CSTF2, CSTF2T and CSTF3 subunits); FLASH, FLICE-associated huge protein; HCC/hCF, histone cleavage complex (SYMPK, CPSF100 and CPSF73 subunits)/human cleavage factor; hPSF, human polyadenylation specificity factor (including CPSF30, CPSF160, WDR33 and FIP1 subunits); PPase, phosphatase module (including SSU72, WDR82 and PP1CA/B/C/R10 subunits); SLBP, stem–loop-binding protein; SYMPK, Symplekin; U7 snRNP, U7 small nuclear ribonucleoprotein (including, besides U7 snRNA, LSM11, LSM10, SNRPD3/B/E/F subunits and the SNRRPG subunit not detected in our analysis).

We designed a purification strategy to capture human 3′-pre-mRNA-processing complexes from nuclear extracts of HEK293-F cells (Figure 1D), relying on the assembly of the endogenous complexes onto the H2A_4m RNA bait before binding on a streptavidin–agarose matrix, and elution using UV irradiation (20). Western blot analysis of the abundance of captured SLBP, CPSF73 and SYMPK was used to optimize the different steps. Addition of recombinant N-FLASH to nuclear extracts, previously reported to promote assembly of the mouse histone mRNA-processing complex (20,24), did not improve the yield of purified human complex (Figure 2A), suggesting the functionality of endogenous human FLASH in our assays. Notably, the binding of SYMPK and CPSF73 was completely abolished by using H2A_4m annealed with U7, but only partially when using aU7. Similar results were obtained by using a different human cell line, HeLa-S3 (Figure 2A).

H4_1m was also able to capture CPSF73/SYMPK/SLBP (Supplementary Figure S1B) and, using SL, both in the absence and presence of aU7, we noticed a slight increase in CPSF73 and SYMPK levels, whereas SLBP disappeared (Supplementary Figure S1C). Additionally, association of the U7 snRNP module of the histone RNA-processing complex with H4_1m was suggested through the presence of Lsm11, which was abrogated by the use of aU7 but not SL (Supplementary Figure S1C). This indicates that H4_1m is able to recruit the CPSF73/SYMPK endonuclease module but, compared with H2A_4m, a smaller fraction is aU7 sensitive and likely involved in the formation of the histone RNA processing complex.

In order to validate that our purification workflow captures functional histone pre-mRNA-processing machinery, we investigated the cleavage activity of a 60-mer double biotinylated wild-type pre-mRNA of H2AC18, monitored by a chemiluminescent-based northern blot assay (44) (Figure 2B). Despite a non-specific degradation during the assays, we clearly detected a major product in the reactions carried out with both crude nuclear extract and the purified samples. In contrast, the same product band was not detected in the presence of aU7/SL RNA. Due to the 5′–3′ exonuclease activity of CPSF73 in the histone mRNA-processing complex (25), we expect that the major product band is generated primarily by the 29-mer 5′-cleavage fragment, rather than the 31-mer 3′ fragment. Indeed, the smear located just below is probably due to the degradation of the 31-mer fragment by CPSF73. In summary, we confirmed that both crude HEK293 nuclear extracts and purified 3′-pre-mRNA-processing complexes are competent for the cleavage of histone H2AC18 pre-mRNA, and that this mechanism is highly dependent on the presence of U7 snRNP.

Altogether, our results demonstrate that H2AC18 pre-mRNA associates with two distinct populations of the CPSF73/SYMPK endonuclease module, one being aU7 sentitive and active in histone pre-mRNA processing, probably as part of the histone pre-mRNA-processing complex (HCC module), and the other being aU7 insensitive.

### H2AC18 pre-mRNA assembles both full histone and canonical pre-mRNA-processing complexes

In the previous section, we described the use of aU7 and U7 snRNA sequences as negative controls to assess the assembly of the different processing complexes. While the first blocks the endogenous U7 snRNA, acting as a short HDE sequence, the second is engaged in a duplex with H2A_4m, spanning the HDE region and two-thirds of the PAS sequence (Figure 1B). As a consequence, and unlike the aU7 sequence, the U7 RNA is thought to disrupt not only HDE-dependent interactions but also all PAS-dependent interactions. Therefore, we hypothesized that the second population of the CPSF73/SMPK endonuclease module, whose binding is aU7 insensitive but sensitive to U7 snRNA —and thus most probably PAS dependent— originates from the assembly of the canonical 3′-end pre-mRNA complex on the H2A sequence.

In order to confirm this hypothesis and, more generally, to determine the composition of the 3′-processing complexes bound to H2A_4m, we performed an MS-based proteomic characterization of the H2A_4m-bound proteins, which revealed, among the most abundant proteins, the presence of all subunits belonging to the histone mRNA-processing complex (except SmG, also undetectable in mouse) (20). Remarkably, we also found all proteins belonging to the CPSF and CstF modules of the canonical pre-mRNA-processing complex (including the variant TauCstF-64/CSTF2T) (Supplementary Figure S2A; Supplementary Table S2), as well as subunits of the CFIm module, the phosphatase module and Clp1 from the CFIIm module. Our experiments did not reveal the presence of the human poly(A) polymerase alpha (PAPOLA), despite its known association with the hPSF module via Fip1 (45). Although in lower abundance compared with 3′ pre-mRNA-processing complexes, we also identified proteins belonging to the spliceosome, the exosome and RNA polymerase II-associated factors. This is in agreement with a previous study (46) and it can be interpreted through the binding of free RNAs —unbound to 3′-pre-mRNA-processing complexes— to other protein–RNA complexes.

Next, we sought to investigate how disrupting the recognition of the whole PAS–HDE region, or the HDE region only, of H2AC18 pre-mRNA impacts the specific assembly of the two mRNA-processing complexes. Thus, we used MS-based proteomics to compare proteins captured by H2A_4m in the absence or in presence of the control molecules U7 snRNA and SL (H2A_4m + U7/SL) or aU7 and SL (H2A_4m + aU7/SL). The addition of exogenous U7 snRNA caused a general decrease in the abundance of both histone and canonical 3′-pre-mRNA-processing complexes, suggesting a general inhibitory effect on complex assembly onto the HDE–PAS region (Figure 2C; Supplementary Figure S2B). On the other hand, the presence of aU7/SL only impacted the subunits of the histone RNA-processing complex, and in particular the U7 snRNP ring and FLASH (besides SLBP, blocked by the synthetic SL), while the canonical pre-mRNA-processing complex was mostly unaffected (Figure 2D; Supplementary Figure S2C; Supplementary Table S2). Interestingly, the subunits of the shared HCC/hCF module (CPSF73, CPSF100 and SYMPK) followed the trend of the canonical complex, and their abundance remained similar to those observed in the H2A_4m sample. This agrees with our western blotting results, which showed only a moderate decrease of CPSF73 and SYMPK bands (Figure 2A). The protein most affected in the presence of U7/SL and aU7/SL was ERI1/3′hExo, a histone mRNA-degrading exonuclease that can form a ternary complex with SLBP and the SL region (47). This result confirms a clear association of ERI1 with H2AC18 pre-mRNA and its role in the regulation of histone pre-mRNA levels. The heterogeneous nuclear ribonucleoprotein U-like 1 (HNRPUL1) was also detected (Supplementary Table S2), which is in agreement with published data showing its involvement in U7 snRNP-dependent transcriptional repression of RD histones (48). Collectively, these findings demonstrate that H2AC18 pre-mRNA can very efficiently capture both human histone and canonical 3′-pre-mRNA-processing complexes.

### Assembly of the canonical 3′-pre-mRNA processing complex on histone pre-mRNAs is blocked by PAS disruption

**Figure 3.**
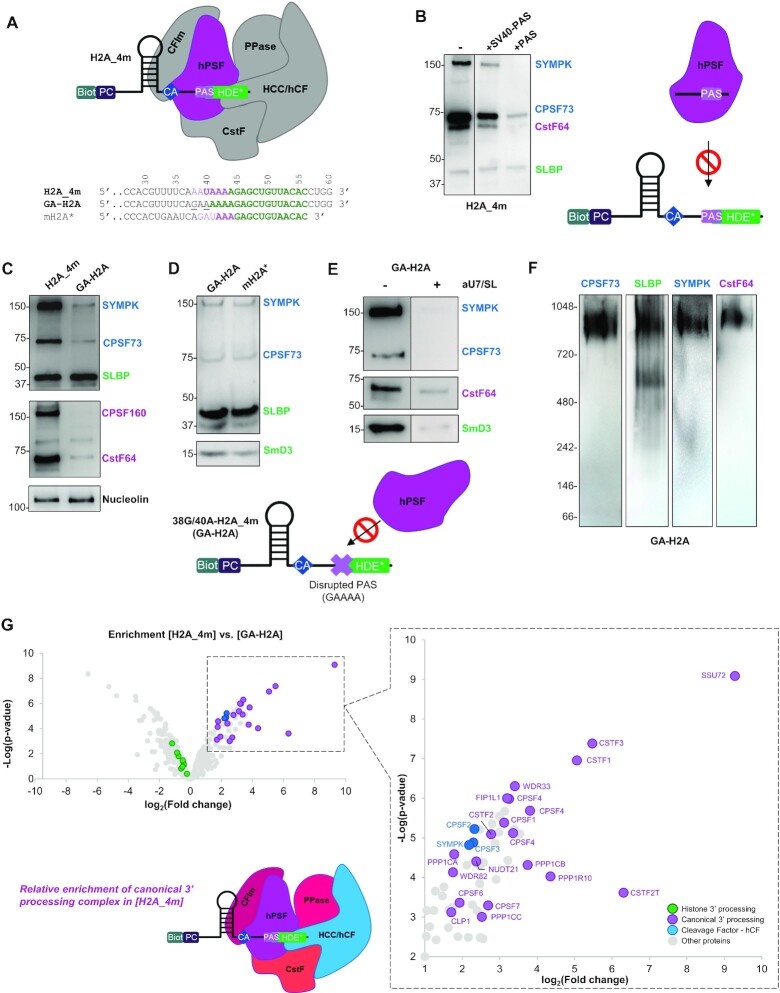
The integrity of the PAS sequence in H2AC18 is essential for the assembly of the canonical 3′-pre-mRNA-processing complex. (**A**) Assembly scheme of the canonical complex on the H2A_4m RNA: in purple is depicted the hPSF module (CPSF73, CPSF100 and SYMPK), mediating PAS recognition in the RNA sequence; elements predicted as not directly involved in the interaction (HDE* and other complex modules) are depicted in gray; a sequence alignment among H2A_4m, its double mutant GA-H2A and the mouse RNA mH2A* is reported below; in the H2A_4m sequence, the PAS is indicated in purple, mutated residues in 38G/40-H2A_4m are underlined and HDEs are indicated in green. (**B**) Western blot analysis of samples purified with H2A_4m bait RNA, in the absence or presence of PAS-containing control RNAs; the use of these molecules allowed blocking, in a selective manner, of the assembly on H2A_4m of the entire canonical complex via the hPSF module (scheme on the right). (**C–E**) Comparison by western blot of processing factor abundances in samples purified with different RNAs/conditions: H2A_4m *versus* GA-H2A (C), GA-H2A *versus* mouse RNA mH2A* (D) and GA-H2A in the absence or presence of aU7/SL RNAs (E); mutations in GA-H2A RNA disrupting the PAS sequence prevent the assembly of the entire canonical complex via the hPSF module (scheme below). In (B) and (E), the lanes separated by a black line come from different zones of the same membrane. In (C), nucleolin is used as a control protein to demonstrate the specificity of the PAS disruption effect on proteins belonging to the canonical mRNA-processing complex. Exposure time in (E) was increased, compared with (D), to better show the difference between samples without or with aU7 + SL (**F**) Western blot analyses with four antibodies of a sample purified with GA-H2A, DTSSP-cross-linked after elution from streptavidin–agarose beads and run in native PAGE. (**G**) Differential analysis of protein abundances in [H2A_4m] *versus* [GA-H2A] samples; relevant identified proteins that are at least 2-fold more abundant in [H2A_4m] samples compared with [GA-H2A] samples are annotated in the zoom-in graphs (subpanel on the left); a schematic representation of complex relative abundance is shown (lower subpanel). Abbreviations: CFIm, cleavage factor 1m module (including NUDT21, CPSF6 and CPSF7 subunits); CPSF, cleavage and polyadenylation specificity factor; CstF, cleavage-stimulating factor module (including CSTF1, CSTF2, CSTF2T and CSTF3 subunits); FLASH, FLICE-associated huge protein; HCC/hCF, histone cleavage complex (SYMPK, CPSF100 and CPSF73 subunits)/human cleavage factor; hPSF, human polyadenylation specificity factor (including CPSF30, CPSF160, WDR33 and FIP1 subunits); PPase, phosphatase module (including SSU72, WDR82 and PP1CA/B/C/R10 subunits).

Because our H2A_4m RNA bait contains a PAS partially overlapping the HDE (Figure 3A), we hypothesized that the assembly of the canonical complex proceeds mainly through the recognition of this sequence by the hPSF module. To test this hypothesis, we performed complex purification either by using H2A_4m in the presence of two different canonical pre-mRNAs —a 40-mer sequence from the simian virus 40 (SV40) late PAS site and a 17-mer PAS-containing RNA (12) (Figure 3B; Supplementary Table S1)— or by using a histone H2AC18 pre-mRNA variant (38G/40A-H2A_4m, herein GA-H2A) containing a disrupted PAS (Figure 3A, C–E; Supplementary Table S1). GA-H2A mutations were designed in the first half of the PAS sequence, in order to preserve the complementarity of the second half —which is also part of the HDE— with the endogenous U7 snRNA. The two mutations disrupted the A38 interaction with CPSF30 (49), and prevented the formation of the Hoogsteen base pair between U40 and A43 in the WDR33-binding pocket (10,12) (Supplementary Figure S3). The use of both canonical PAS sequences as assembly inhibitors and of the GA-H2A RNA consistently resulted in a drop of purified SYMPK and CPSF73, while leaving SLBP unaffected. The purification of CstF-64 was also substantially affected, but not completely abolished (Figure 3B, C), confirming partial association of CstF-64 with the histone-processing complex (22).

As SYMPK and CPSF73 belong to both histone and canonical complexes, we continued the investigations with GA-H2A and analyzed by western blot the presence of two additional proteins, CPSF160 and SmD3, belonging, respectively, to the canonical hPSF and the histone U7 snRNP complex. As expected, CPSF160 binding was totally abolished by the disruption of the PAS site of H2AC18 pre-mRNA (Figure 3C), whereas the purification of the histone complex subunit SmD3 was unaffected (Figure 3D). Thus, the efficiency of GA-H2A in capturing the SmD3 subunit of the histone mRNA complex, as well as SYMPK, CPSF73 and SLBP, was comparable with that of the mouse pre-mRNA mH2a* (Figure 3D). The latter indeed contains an alternative PAS motif, GAUAAA (Figure 3A; Supplementary Figure S1A,B), which is recognized with low affinity by the hPSF module *in vitro* (49). Conversely, binding of the SYMPK, CPSF73, SmD3 and CstF-64 subunits to GA-H2A was strongly affected by the incubation with aU7 (Figure 3E). Altogether, these results confirm that the association of these four proteins with human H2AC18 pre-mRNA is U7 snRNP dependent, and corroborates previous findings on the mouse processing machinery obtained with mH2A* RNA (20).

Similarly, we investigated the ability of the PAS located upstream of the SL of H4C11 to recruit the hPSF module of the canonical complex. Notably, this PAS overlaps the first 2 nt of the AAA motif which precedes the SL and is part of the SLBP binding site (47). Therefore, we reasoned that, unlike H2AC18 PAS, which is positioned well downstream of the SL and cannot interfere with SLBP binding, H4C11 PAS may generate a competition between SLBP and the hPSF module. CPSF160 is able to associate with H4_1m (Supplementary Figure S1C), and the binding of this canonical factor and a major fraction of the CPSF73/SYMPK endonuclease module is PAS dependent, as demonstrated with the 2G/4A variant of H4_1m (2G/4A-H4_1m, herein GA-H4) (Supplementary Figure S1A, C, D). As with GA-H2A, the residual binding of the endonuclease module to GA-H4 is disrupted in the presence of aU7/SL, the same tendency being observed with Lsm11. The addition of SL RNA to nuclear extracts did not influence the levels of CPSF160 bound to H4_1m. However, as previously mentioned, it caused a slight increase in the level of hCF proteins, and a substantial increase in SLBP, purified with GA-H4 (Supplementary Figure S1C). This partially agrees with previous findings (47) and suggests that, in the presence of the hPSF bound to the PAS motif of H4C11 RNA, the binding of SLBP may be perturbed. Overall, these findings support the assembly of canonical and histone pre-mRNA-processing complexes onto H4C11 RNA. Despite this evidence, it has not yet been elucidated in which cellular context(s) this PAS is functional, and polyadenylated transcripts of H4C11 have only been found in SLBP-depleted cells (50), but not in terminally differentiated tissues (33).

To estimate the molecular weight of the histone pre-mRNA-processing complex assembled on H2A pre-mRNA, samples purified with GA-H2A were cross-linked and analyzed by western blotting after native PAGE migration (Figure 3F). Staining with SYMPK, CPSF73 and CstF-64 primary antibodies consistently yielded a single band between 700 kDa and 1 MDa, while the anti-SLBP antibody revealed an additional lower molecular weight band (∼600 kDa). The apparent mass of the major band corresponds to the predicted mass of the complete histone 3′-RNA-processing complex (∼1 MDa), while the second band specifically revealed by the anti-SLBP antibody could be attributed to a U7 snRNP–SLBP–GA-H2A subcomplex probably bound to a FLASH dimer (overall ∼600 kDa), whose N-termini may form a heterotrimer with the Lsm11 subunit of U7 snRNP (35).

The distribution of protein abundances estimated by MS signals in samples purified with GA-H2A (Supplementary Figure S2D; Supplementary Table S2) showed that subunits of the histone processing complex, but not the canonical processing complex, ranked as the most abundant proteins, particularly SLBP and the U7 subunits SmD3/E/F and Lsm10. The comparison of protein abundances in samples using H2A_4m and GA-H2A as baits (Figure 3G; Supplementary Table S2) revealed a drastic decrease in all the subunits belonging to the canonical 3′-pre-mRNA-processing complex, as a consequence of PAS disruption. The abundance of histone mRNA complex subunits (FLASH, SLBP and all Sm and Lsm proteins) remained similar in both samples, with the exception of ERI1, for which an increase of >2-fold with GA-H2A was observed. Importantly, while the abundance of hPSF subcomplex-related proteins (CPSF160, CPSF30, WDR33 and Fip1) shows an ∼10-fold decrease with GA-H2A, the decrease is about half for hCF subunits, suggesting that the remaining fractions of CPSF73, CPSF100 and SYMPK remain bound onto the histone pre-mRNA-processing complex. Interestingly, most CstF subunits (CstF-50, CstF-77 and TauCstF-64) show a >30-fold decrease with GA-H2A, except CstF-64 with a 7-fold decrease, consistent with our western blot results and further highlighting the involvement of CstF-64 in the histone RNA-processing complex. In conclusion, the association of the canonical 3′-processing complex with the H2AC18 pre-mRNA is PAS dependent, and disruption of this sequence element prevents the assembly of this complex onto the human histone H2AC18 pre-mRNA.

### H2AC18 mRNA associates with the canonical and histone 3′-end mRNA processing complexes in a mutually exclusive manner

Due to the distinctive arrangement and close proximity of the PAS and HDE sequence elements in H2AC18 mRNA, and because of the molecular dimensions of the canonical and histone mRNA-processing complexes, it is plausible that binding of one complex to the H2AC18 pre-mRNA, thus occupying the entire PAS-HDE region, prevents assembly of the other complex, and vice versa. We have worked on this hypothesis both by structural modeling and by using biochemical approaches.

**Figure 4.**
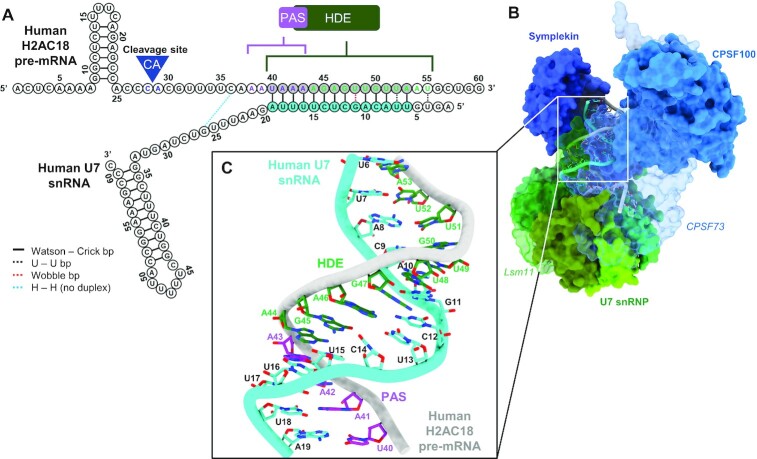
Structural model of the histone 3′-end pre-mRNA-processing machinery in complex with human H2AC18 mRNA. (**A**) 2D structures of wild-type H2AC18 pre-mRNA and U7 snRNA, with their base-pairing residues; the HDE and U7 regions involved in duplex formation and shown in the H2AC18 complex model (C) are depicted in gray and blue, respectively. (**B**, **C**) 3D structural model of the core processing machinery, in complex with H2AC18 pre-mRNA (overall view) (B) and zoomed-in view on the pre-mRNA duplex region with U7 snRNA (C). The model was built starting from the EM structure of the reconstituted human histone pre-mRNA processing machinery, in complex with mouse H2a RNA (PDB ID: 6V4X) (22) In (B), Lsm11 (in U7 snRNP) and CPSF73 are depicted as semi-transparent to allow visualization of RNAs. In (C), the four H2AC18 PAS residues involved in the base pairing are indicated with purple labels, while the other HDE residues are indicated in green. A more detailed representation of the structural differences between our model of the core complex and the reference deposited structure is shown in Supplementary Figure S4.

In the recent structure of the recombinant human holo-U7 snRNP (22) (PDB ID: 6V4X), the duplex between the mouse mH2a HDE and the human U7 snRNA 5′ end extends into the machinery core with 12 bp and a U–U pair. Based on this structure, we have built a model of U7 snRNP in complex with human H2AC18 mRNA (Figure 4A–C; Supplementary Figure S4A), which shows a longer HDE with two additional Watson–Crick pairs (Supplementary Figure S4B, C) compared with mouse mH2A* (Supplementary Figure S4B, D), involving U40 and A41 (Figure 4C; Supplementary Figure S3). In our model, two-thirds of the PAS sites are involved in base pairing with U7 snRNA. Consequently, this PAS sequence would not be available for recognition by the canonical hPSF module (CPSF30, CPSF160, WDR33 and Fip1). This is in agreement with the western blot and MS results showing that the addition of U7 snRNA alone (not in complex with the heptameric U7 Sm ring) is sufficient to hinder the assembly of the canonical processing complex. Of note, there are no other canonical PAS sequences downstream of the HDE in the 3′-UTR of H2AC18, and the position of this H2A cleavage site for polyadenylation, described by previous works (33,43), is compatible with the regulation by the PAS sequence discussed here.

**Figure 5.**
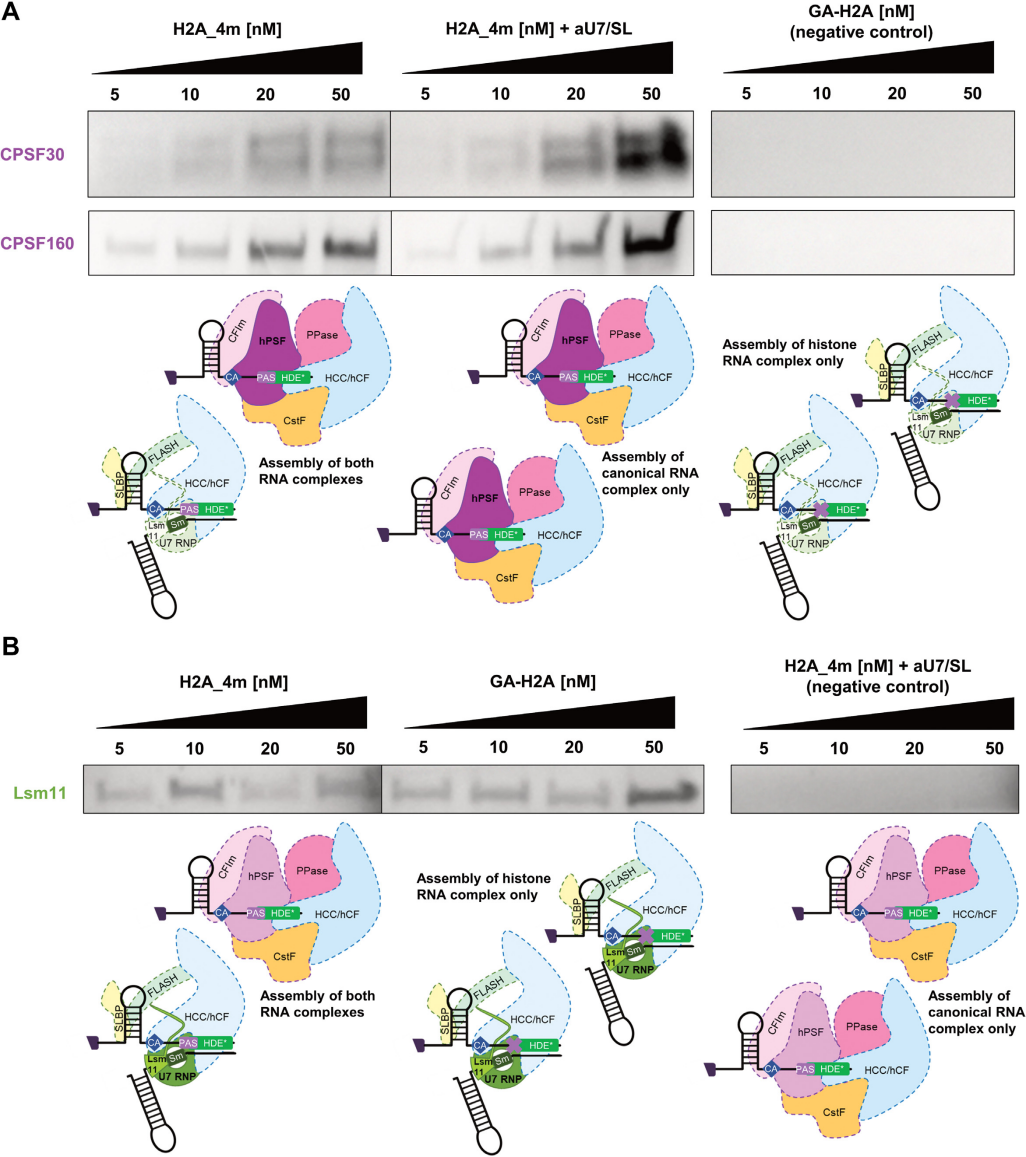
Assembly of histone and canonical mRNA processing complexes on human H2AC18 pre-mRNA is mutually exclusive. Samples were purified from HEK nuclear extracts in different conditions, using increasing concentrations of H2A_4m, GA-H2A or H2A_4m in the presence of aU7/SL (the two control RNAs were used at a constant concentration). The abundances of human polyadenylation specificity factor (hPSF) CPSF30 and CPSF160 proteins (**A**) and of Lsm11 from the U7 snRNP (**B**), belonging to the canonical and histone mRNA processing complexes, respectively, were monitored by western blot. In each panel, the schematic shows the purified complexes for each condition, and the monitored complex modules are highlighted with solid fill colors and bold characters, while the other modules are shown with lighter colors and dashed contour lines. H2A_4m/H2A_4m + aU7/SL samples (A) and H2A_4m/GA-H2A samples (B) were prepared in parallel, run on the same gel and imaged at the same time, in order to facilitate the comparison between each set of samples.

Subsequently, in order to experimentally establish whether the assembly of the two processing complexes on H2AC18 is mutually exclusive, we set up a competition assay and monitored variations in the abundance of protein subunits belonging exclusively to either the histone mRNA complex or the canonical mRNA-processing complex. The relative abundance of bound proteins was measured in samples in which the assembly of each of the two complexes was alternately inhibited. For this, we monitored by western blot the hPSF-specific CPSF160 and CPSF30 subunits for the canonical complex, and the U7 snRNP-specific subunit Lsm11 for the histone complex. This was done in samples purified with increasing concentrations of H2A_4m, GA-H2A and of H2A_4m in the presence of a constant [aU7/SL] (Figure 5). As expected, the binding of the two CPSF subunits is totally abolished with GA-H2A, and Lsm11 is not detected in the presence of the aU7 oligonucleotide. Moreover, hPSF proteins are more abundant in H2A_4m + aU7/SL samples (i.e. when assembly of the histone mRNA processing complex is inhibited) than in H2A_4m samples, an effect that was more pronounced at higher concentrations of H2A_4m (20–50 nM). Conversely, Lsm11 is more abundant in the GA-H2A sample (i.e. when assembly of the canonical mRNA processing complex is inhibited) than in the H2A_4m sample, in the same concentration range. It must be noted that the observed differences of Lsm11 over the different conditions are modest, and this is probably due to the challenge of monitoring histone mRNA processing factors, which are in lower abundance compared with canonical factors.

Taken together, these observations support a model of assembly of the two 3′-end pre-mRNA-processing complexes onto H2AC18 that is mutually exclusive.

### The H2AC18 PAS sequence is recognized by the canonical 3′-end processing complex in human hMSCs, in terminally differentiated adipocytes and in mouse liver tissue

Polyadenylated mRNA transcripts of H2AC18 and several RD H2B genes were described in several human tissues, as well as in adipocytes generated from terminal differentiation of mouse embryonic fibroblasts (33). In addition, the same RD H2B mRNAs were polyadenylated as a result of adipogenic and osteogenic differentiation in hMSCs (51). Therefore, we sought to investigate the assembly on H2AC18 of pre-mRNA-processing complexes in some of these cells, specifically hMSCs and hMSC-derived adipocytes (Supplementary Figure S5A, B). By incubating H2A_4m with nuclear extracts from hMSCs, we were able to capture the CPSF73/SYMPK of the endonuclease module hCF, and CPSF160 from the hPSF module (Supplementary Figure S5C). However, some proteins belonging specifically to the histone pre-mRNA-processing complex (such as SLBP and Lsm11) remained undetected even when raising the concentration of H2A_4m to 1 μM. The western blot results were mostly confirmed by MS analysis of the H2A_4m purified sample, which detected many of the proteins belonging specifically to the canonical processing complex (especially CPSF160/CPSF1, Cstf50/CSTF1 and Cstf77/CSTF3), all the proteins of the shared endonuclease module, but only a few proteins associated with histone mRNA processing, mostly U7 Sm subunits and ERI1 (Supplementary Figure S5D; Supplementary Table S3). Moreover, interactions between proteins of the hCF/hPSF modules and H2A_4m were found to be mainly mediated by the PAS region, as shown by the western blot results with the GA-H2A RNA (Supplementary Figure S5C). Western blot and MS analyses of samples prepared using H2A_4m and nuclear extracts from terminally differentiated adipocytes (Supplementary Figure S5B) did not yield significant differences from hMSC samples, mostly showing association of H2A_4m with proteins belonging to the hPSF/hCF/Cstf modules (Supplementary Figure S5C, D; Supplementary Table S3).

Considering the high degree of conservation of the PAS sequence and recognition mechanism in mammals, and the presence of poly(A) transcripts of Hist2H2AA3 in mouse liver (33), we tested the ability of human H2AC18 RNA to promote pre-mRNA-processing complex assembly in nuclear extracts from a fully differentiated tissue, i.e. mouse liver. H2A_4m was able to recruit proteins from the endonuclease module (Cpsf73 and Sympk) and Cpsf160, and their recruitment was PAS dependent (Supplementary Figure S5E). Results from the MS analysis of the H2A_4m and GA-H2A purified samples were in agreement with the western blot results, although no peptides belonging to mouse Sympk were detected. Moreover, MS results obtained with the H2A_4m sample revealed the assembly of proteins from the Cstf module, from FIP and from the U7 snRNP module of the histone RNA-processing complex (Supplementary Figure S5F; Supplementary Table S4), which were not detected in the GA-H2A sample.

In conclusion, our findings showed that the PAS sequence of histone H2AC18 pre-mRNA can recruit the canonical pre-mRNA-processing complex in both human MSCs and MSC-derived adipocytes, as well as in mouse liver tissue. Curiously, we were not able to detect a fully assembled histone pre-mRNA-processing complex in hMSC extracts, most probably due to the low abundance of some histone-processing factors and/or to a small population of cells in S phase at the time of sample preparation.

## DISCUSSION

Histone mRNA transcription and processing take place in the histone locus bodies (HLBs), which are specific, core–shell-arranged nuclear compartments assembled at histone loci (52), and result from the concentration, spatial organization and functional coordination of all the factors necessary for pre-mRNA transcription and cleavage, including the transcriptional coactivator NPAT, the RNA polymerase II, FLASH and the whole histone mRNA-processing machinery (6–7,52,53). NPAT is crucial for HLB integrity and it is involved in the transcription of histone mRNAs along with other factors also used for transcription of canonical polyadenylated mRNAs (30). It is required for entry into S phase, and its phosphorylation during this phase is essential for histone gene expression (54). NPAT is also important for the recruitment of FLASH (55,56), which is involved in histone pre-mRNA processing (57) as a component of the holo-U7 snRNP complex (20,22,24).

Human 3′-pre-mRNA processing relies on bipartite *cis*-acting sequence elements: the PAS/DSE sequences regulating the processing of canonical pre-mRNAs into cleaved and polyadenylated transcripts, and the SL/HDE sequences promoting the cleavage of RD histone pre-mRNAs (7). While the cleavage and polyadenylation machinery is functional during all phases of the cell cycle, the active holo-U7 snRNP processing machinery is present in the HLBs only during the S phase (58), and histone mRNA processing is tightly cell cycle regulated (6,7,30).

Thus, the entire RD histone mRNA biogenesis is regulated in terms of RNA sequence, nuclear localization and cell cycle phase. This yields mature histone mRNA transcripts that terminate in an SL motif —fundamental for proper transcript disposal at the end of DNA replication— which associates with SLBP to provide mRNA stability, nuclear egress, translation and degradation.

**Figure 6.**
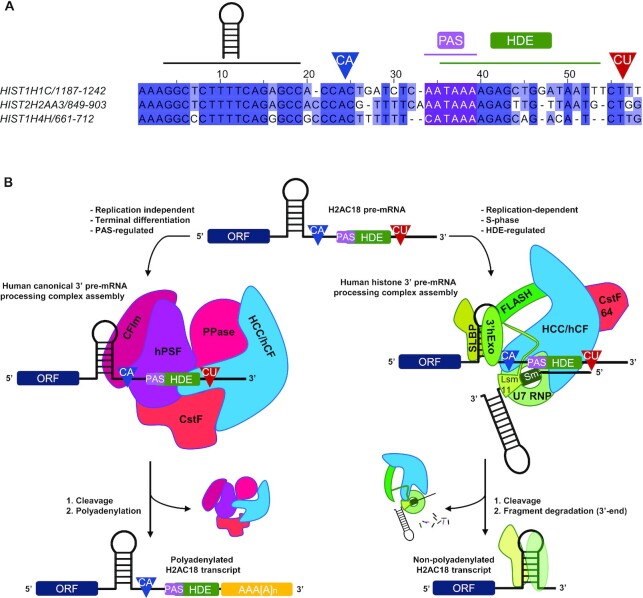
Mutually exclusive complex assembly on wild-type H2AC18 pre-mRNA leads to different processing fates. (**A**) Alignment of 3′-UTR sequences from three RD histone genes which are polyadenylated in terminally differentiated fibroblasts (33) and whose pre-mRNAs contain overlapping PAS–HDE *cis*-acting elements. Above the alignment are indicated the main sequence elements, including the SL hairpin, the two cleavage sites regulated by the HDE (CA cleavage site, in blue) or by the PAS (CU cleavage site, in red) and the position of the PAS–HDE superposition. The three PAS sequence elements are colored in purple. The alignment was generated and modified with Jalview (74). (**B**) Model for the mutually exclusive complex assembly on H2AC18. The pre-mRNA 3′-UTR supports the formation of either the replication-dependent histone mRNA-processing complex (regulated by the HDE during S phase) or the replication-independent canonical 3′-processing complexes in specific terminally differentiated tissues (regulated by the PAS region), leading to non-polyadenylated or polyadenylated mature RNA transcripts, respectively.

However, remarkable exceptions of RD histone genes expressed as polyadenylated transcripts in specific biological contexts have been reported. In particular, a subset of 10 polyadenylated RD histone transcripts was identified in human tissues (33), seven of which retained the SL and the HDE. The members of the H2A and H2B families belonging to this subset, and their mouse orthologs, are expressed as polyadenylated transcripts in terminally differentiated fibroblasts, likely to replace damaged histones in non-dividing conditions (33,51). HIST1H2BC and HIST1H2BD pre-mRNAs lose their SL and HDE motifs following splicing, and are processed at a poly(A) site located downstream of the 3′-splicing site. On the other hand, poly(A) mRNA transcripts of HIST1H1C and HIST2H2AA3 retain the SL/HDE motifs, including the HDE-overlapping PAS sequence (Figure 6A), which supports cleavage and polyadenylation of HIST1H1C and HIST2H2AA3 upon terminal differentiation (33) or knockdown of key RD histone 3′-end pre-mRNA processing factors (50,59). Interestingly, the HIST1H4H pre-mRNA contains a PAS sequence (CAUAAA) overlapping the HDE 5′ end (Figure 6A) and, although polyadenylation was mainly found in human tissues at a distal site well downstream of the PAS–HDE superposition (33), an additional poly(A) site has been described in HIST1H4H (60), the position of which is compatible with regulation by the upstream CAUAAA PAS.

We have undertaken the study of this intriguing structure of human RD histone pre-mRNAs and chosen to focus on H2AC18, as an archetype of this distinctive sequence architecture. We formulated a first fundamental question: how can H2AC18 pre-mRNA assemble both 3′-end canonical and histone mRNA-processing complexes? We demonstrate that the two complexes are recruited by H2AC18 mRNA in a mutually exclusive manner, as they cannot bind the same RNA molecule at the same time. Moreover, our findings have double relevance: first, by using pre-mRNAs containing 3′-UTRs of H2AC18, we were able to purify an endogenous and functional human histone mRNA-processing complex from several different human cells (Figure 1), thereby complementing previous findings on the mouse machinery (20). Second, and more strikingly, we show that H2AC18 pre-mRNA captures very efficiently the human canonical 3′-end-processing machinery (Figure 2), and that, after PAS disruption, the assembly of this complex, but not of the histone-processing complex, is selectively blocked (Figure 3).

Associated with H2AC18, we identified all the subunits essential for histone mRNA processing (22). Moreover, our results corroborate previous findings suggesting an association of ERI1/3′hExo with the holo-U7 snRNP complex and histone pre-mRNA before endonucleolytic cleavage (61). ERI1 is a 3′–5′ endonuclease involved in degradation of several RNAs, including histone mRNAs (62,63), and it cooperatively associates with SLBP in a ternary complex with the SL motif of histone mRNAs (47,64). During the S phase, there is an equilibrium between degradation by ERI1 and uridylation by the terminal uridyl transferase 7 (TUT7) of the 3′-ACCCA tail, which is located in processed mRNAs immediately after the SL element required for SLBP binding; this ensures mRNA protection from decay and correct translation (65). However, this equilibrium is disrupted at the end of the S phase, when ERI1 degrades part of the 3′-stem region and dissociates from the SL, triggering the initiation of mRNA decay —concomitantly with SLBP dissociation and SL deprotection— via uridylation and degradation by the exosome (6,62,63).

We found subunits of the majority of known modules belonging to the canonical pre-mRNA-processing complex. Notably, the abundance of TauCstF-64 on H2AC18 pre-mRNA was found to be more affected than that of CstF-64 by PAS disruption, suggesting a preferential association of the former with the canonical processing complex. On the other hand, we have found CstF-64 associated with both processing complexes, consistent with previous hypotheses about the different roles of CstF-64 and TauCstF-64 in 3′-pre-mRNA processing (21,66).

Additionally, we show how a U7 snRNA oligonucleotide can not only block the assembly of the histone complex on H2AC18, but can also hinder the formation of the canonical 3′-end-processing complex on the PAS region. The competition experiments with GA-H2A and aU7/SL RNA (Figure 5), together with our structural models (Figure 4;Supplementary Figure S4), support a mechanism in which the assembly of the two 3′-end-processing complexes on H2AC18 is mutually exclusive. Finally, by investigating complex assembly in nuclear extracts from undifferentiated hMSCs, terminally differentiated adipocytes and mouse liver tissues (Supplementary Figure S5), we could conclude that H2AC18 mRNA is able to recruit, in a PAS-dependent manner, endogenous canonical mRNA-processing complexes of primary cells and of mammalian tissues, thereby supporting and extending previous findings on the polyadenylation of RD histone genes (32,33).

We also studied complexes associating with the H4C11 pre-mRNA, as another example of RD histone RNA containing an unusual PAS sequence, in this case located in the region immediately upstream of the SL (Supplementary Figure S1). Interestingly, H4C11 mRNA was previously found to be polyadenylated in human osteosarcoma cells, following knockdown of SLBP (50), but not polyadenylated in other studies (32,33). Here we found that the 3′ end of H4C11 pre-mRNA was able to recruit both histone and canonical RNA-processing complexes, and that the recruitment of the latter was mediated by the PAS sequence upstream of the SL. This shows that the localization of the PAS sequence in the proximity of the hairpin motif still allows assembly of the canonical complex, although the presence of SLBP and SLBP-associated factors involved in H4C11 mRNA metabolism may prevent assembly in specific nuclear environments, explaining the absence of polyadenylation in some cells (32).

Based on our findings, it is tempting to speculate on the cellular mechanism controlling the switch between the two 3′-end-processing modes —leading to the two distinct polyadenylation states of H2AC18 mRNA— and the fate of the two different mRNA transcripts. We propose a model (Figure 6B) in which, during the S phase, the HLB hosts and coordinates histone mRNA transcription and processing initiated by phosphorylated NPAT, the recruited FLASH and the other histone pre-mRNA-processing factors. Thus, the nascent mRNA, synthesized by RNA polymerase II, is readily recognized by SLBP on the SL 5′ side, which facilitates assembly of the holo-U7 complex on the HDE/PAS region and processing. After cleavage, the mature mRNA, ending with a 3′ SL, is released and ready for nuclear export and translation. At this point, SLBP, still bound to the 5′ side of SL, plays an equivalent role to the poly(A) tail of canonical mRNAs, ensuring mRNA transport (67), translation (68) and degradation (69,70).

Outside the S phase and in specific cellular contexts, such as cell terminal differentiation, the need for new histone proteins cannot be met by the usual cell cycle-dependent regulatory mechanisms, as many of the factors needed for histone gene transcription and processing are not active (NPAT, U7 snRNP), not enriched in the HLBs (FLASH) or low in abundance (SLBP) (7). An alternative possibility for the processing of RD histone pre-mRNAs would be the recruitment of the canonical pre-mRNA-processing complex on valid PAS sequences to synthesize poly(A) transcripts. Lyons *et al.* postulated the presence of a tight regulatory system for the transcription and processing, outside the S phase, of low levels of polyadenylated histone mRNAs, which are further controlled, in the case of histone mRNAs containing an intron in the 3′-UTR, by nonsense-mediated decay (NMD) (33). Furthermore, the distance between the termination codon and the polyadenylate-binding protein 1 (PABPC1), which binds to the poly(A) tail and impacts translation, dramatically affects mRNA stability and can lead to NMD of excessively long 3′-UTRs (71). Based on these considerations, we hypothesize that the positioning of the PAS in a region near the SL (Figure 6A), which represents a ‘marker’ for an optimal distance from the stop codon (72), may lead to the expression of more stable polyadenylated histone transcripts used by terminally differentiated cells to produce histone proteins.

However, how would overlapping PAS/HDE sequences promote mRNA processing by the histone complex over the canonical complex? If one considers that several poly(A) histone mRNAs are expressed at low levels during the S phase (32), an idiosyncratic PAS/HDE architecture leading to a mutually exclusive processing complex assembly, such as the one found in H2AC18, may be a mechanism to limit processing by the canonical complex and to favor the 3′-end processing by the holo U7 complex in the HLB, where the cell cycle-dependent histone mRNA-processing factors are concentrated (53). Another possibility is that the presence of overlapping PAS/HDE sequences in the pre-mRNA architecture may represent a ‘fail-safe’ mechanism to produce poly(A) histone transcripts, in case factors associated with the U7-dependent mechanism would be disrupted, which is in agreement with what has been observed for H2AC18 and histone H1C (HIST1H1C) (50,59).

Collectively, our results show that human RD H2AC18 uses a distinctive mechanism to regulate its 3′-pre-mRNA processing by selectively recruiting either the canonical or the histone processing complex (Figure 6B). We found that, by overlapping these two sequence elements in the 3′-UTR, H2AC18 pre-mRNA deploys a novel mechanism that fully supports the mutually exclusive assembly of the histone and canonical pre-mRNA-processing complexes. This has implications for dictating the polyadenylation fate of this histone pre-mRNA, and future studies will help to further understand the biological role of these PAS/HDE *cis*-regulating elements, not only in H2AC18 but also in other RD histone pre-mRNAs.

## DATA AVAILABILITY

MS data have been deposited at the ProteomeXchange Consortium via the PRIDE partner repository (73) with the dataset identifiers PXD027636 and PXD034984, and are publicly available as of the date of the publication.

## Supplementary Material

gkac878_Supplemental_Files

## Notes

Present address: Domenico Iuso, Faculty of Veterinary Medicine, University of Teramo, 64100 Teramo, Italy.
