# Supplementary material for: Human histone pre-mRNA assembles histone or canonical mRNA-processing complexes by overlapping 3′-end sequence elements
Source: Nucleic Acids Res. 2022 Nov 30;50(21):12425–43. doi: 10.1093/nar/gkac878 (PMC9756948; doi:10.1093/nar/gkac878)
Supplement: gkac878_Supplemental_Files [file gkac878_supplemental_files.zip › SuppMaterials_Ielasi_et_alNAR-Revision2_corrected.pdf]

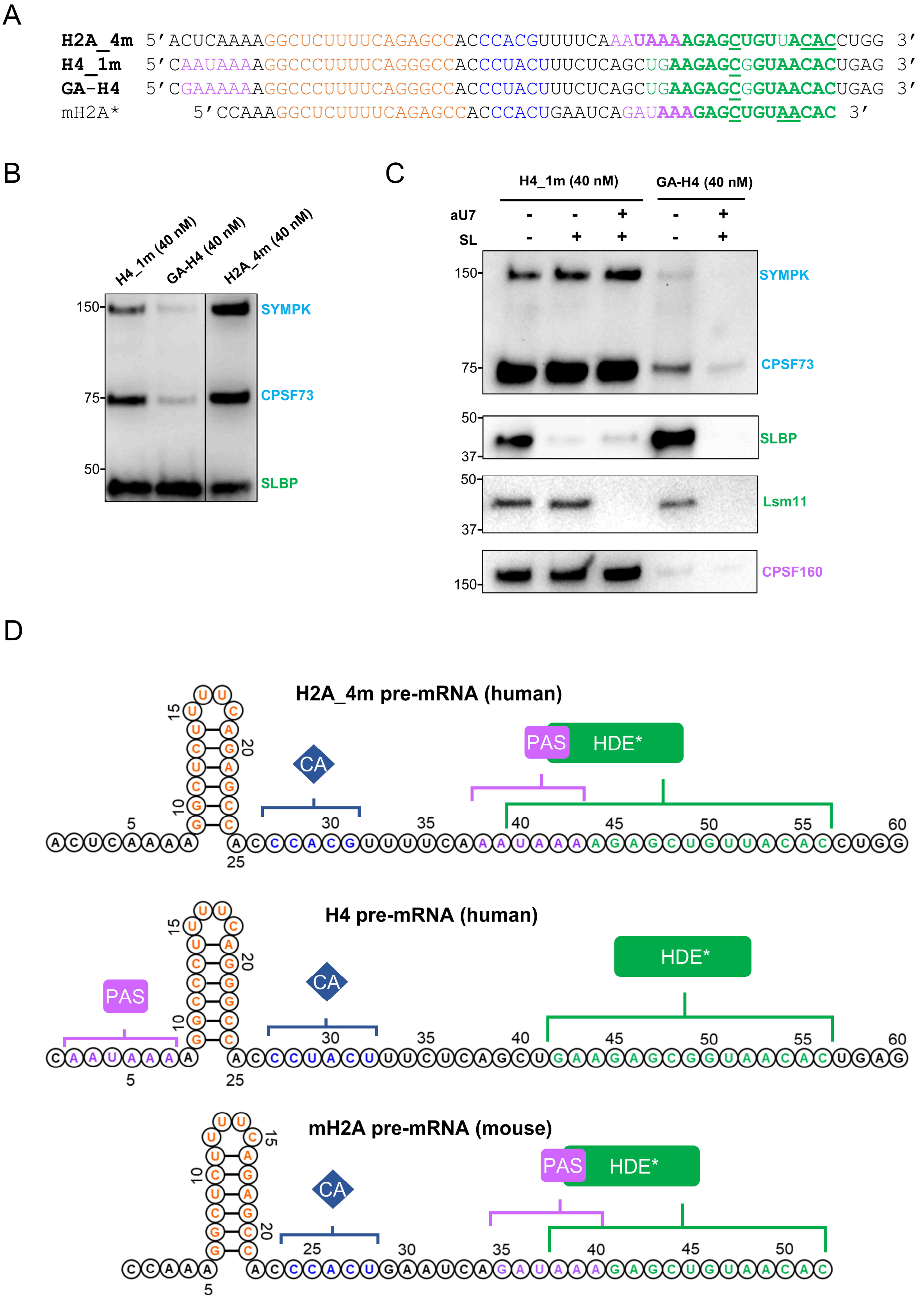
A
H2A_4m 5′ACUCAAAAGGCUCUUUUCAGAGCCACCCACGUUUUCAAAUAAAAGAGCUGUUACACCUGG 3′
H4_1m 5′CAAUAAAAGGCCCUUUUCAGGGCCACCCUACUUUCUCAGCUGAAGAGCGGUAACACUGAG 3′
GA-H4 5′CGAAAAAAGGCCCUUUUCAGGGCCACCCUACUUUCUCAGCUGAAGAGCGGUAACACUGAG 3′
mH2A* 5′CCAAAGGCUCUUUUCAGAGCCACCCACUGAAUCAGAUAAAGAGCUGUAACAC 3′
B
H4_1m (40 nM)
GA-H4 (40 nM)
H2A_4m (40 nM)
150
75
50
SYMPK
CPSF73
SLBP
C
H4_1m (40 nM)
GA-H4 (40 nM)
aU7
SL
150
75
50
37
SYMPK
CPSF73
SLBP
Lsm11
CPSF160
D
H2A_4m pre-mRNA (human)
PAS
HDE*
CA
H4 pre-mRNA (human)
mH2A pre-mRNA (mouse)

Figure S1 – Structural differences between human H2AC18, H4C11 and mouse H2A mRNA 3'-ends, and binding of canonical and histone 3'-end processing factors to human histone H4 and H2 pre-mRNAs. Related to Figure 1 and Figure 2. (**A**) Alignment of pre-mRNA sequences: H2A_4m, H4_1m, GA-H4 (used in this work) and mH2A* (from Sun et al., Science, 2020). Stem loop motifs are indicated in orange, cleavage sites modified with 2'O-methylated nucleotides in blue, PAS motifs in purple and HDE sequences in green. Base-paring residues are indicated in bold. (**B**) Western blot (WB) analysis of samples purified with H4_1m, its variant sequence GA-H4 (containing a PAS-disrupted GAAAAA sequence) and a sample purified with H2A_4m for comparison. (**C**) WB analysis of samples purified with H4_1m and GA-H4, in absence or presence of antisense U7 (aU7) and stem loop (SL) control molecules (**D**) Full architecture of H2A_4m, H4_1m, GA-H4 RNAs, and mH2A* RNA.

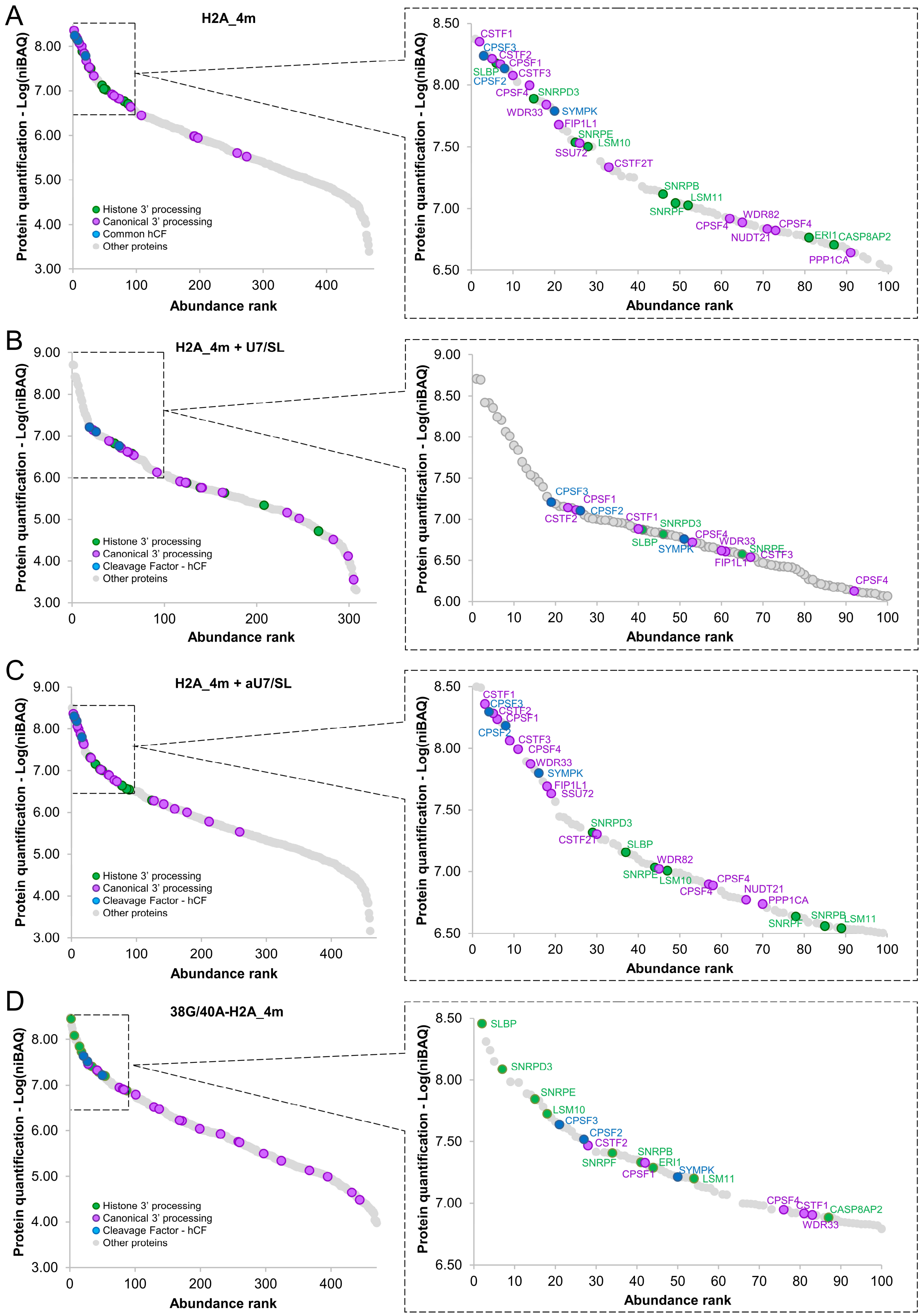

A
H2A_4m
B
H2A_4m + U7/SL
C
H2A_4m + aU7/SL
D
38G/40A-H2A_4m
Protein quantification - Log(niBAQ)
Abundance rank
Histone 3' processing
Canonical 3' processing
Common hCF
Cleavage Factor - hCF
Other proteins
CSTF1
CPSF3
CSTF2
CPSF1
SLBP
CPSF2
CSTF3
CPSF4
SNRPD3
WDR33
SYMPK
FIP1L1
SNRPE
LSM10
SSU72
CSTF2T
SNRPB
LSM11
SNRPF
WDR82
NUDT21
ERI1
CASP8AP2
PPP1CA

Figure S2 – Abundance-based ranking of proteins quantified by MS in samples purified with H2A_4m and GA-H2A_4m pre-mRNAs. Related to Figure 2 and Figure 3. (**A-B-C-D**) Distributions of abundances, expressed as Log_{10}(normalized iBAQ values), of individual proteins detected in samples purified using H2A_4m, alone (**A**) or in presence of U7/SL (**B**) or aU7/SL (**C**), and using GA-H2A (**D**). Identified subunits from canonical and histone 3'-end processing complexes are highlighted. In each panel, an annotated zoom-in plot shows the first 100 proteins of the ranking. H2A_4m (panel A) is representative of 3 different experiments. For a full list of the identified proteins and their ranking according to iBAQ values, see also Table S2.

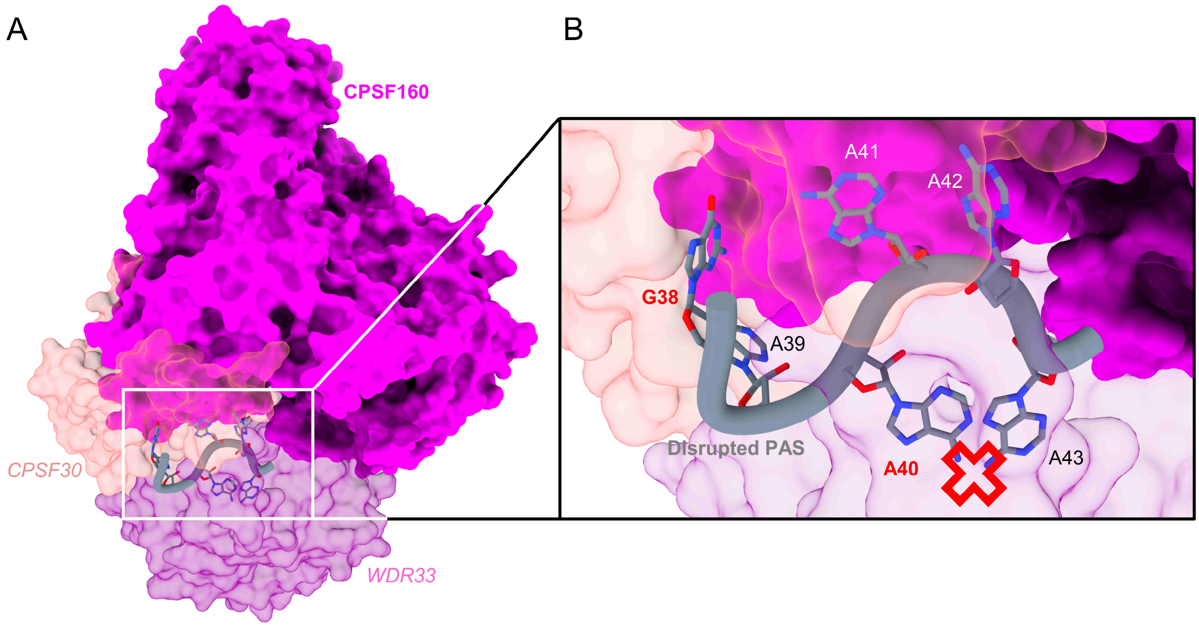

Figure S3 – Structure-based design of mutations in the PAS of human H2AC18 pre-mRNA preventing the assembly of the canonical PSF module. Related to Figure 3**.** (**A**) Overall view of the human PSF module formed by CPSF160/CPSF30/WDR33 (PDB: 6FUW; Clerici et al., 2018) and the PAS region of H2AC18 pre-mRNA with the two mutations introduced to block the assembly into the PSF module (GA-H2A). Semi-transparent subunits (indicated in italic) allow for visualization of the pre-mRNA sequence within the protein complex. (**B**) Zoomed-in view onto the disrupted PAS of the model; please note that the U40A mutation results in a clash between A40 and A43 and is incompatible with the formation of the Hogsteen base pair, essential for the interaction with the PSF module (in agreement with Hamilton et al., RNA, 2019)

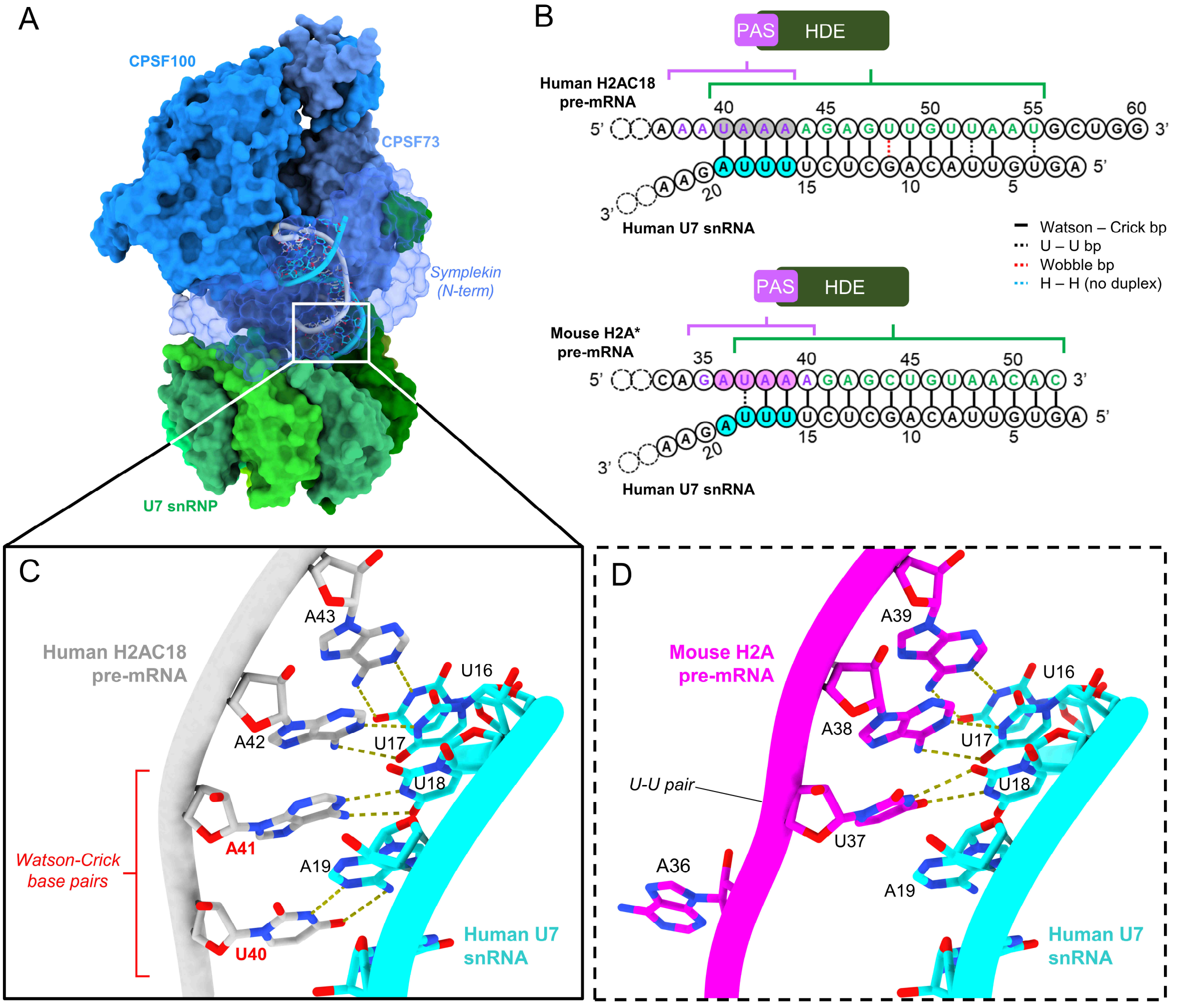

Figure S4 – Model of the human histone mRNA 3' processing machinery, in complex with the human H2AC18 pre-mRNA. Related to Figure 4. (**A**) Overall structure of the core histone 3' pre-mRNA processing complex bound to the 3'-UTR of H2AC18 pre-mRNA, containing the CA cleavage site, the PAS sequence and the HDE region; the latter is involved in an RNA duplex with the human U7 snRNA. The N-terminus of symplekin (indicated in italic) is depicted as semi-transparent to allow visualization of the RNA duplex (see panel C). (**B**) Sequence architecture of the PAS-HDE region of human H2AC18 pre-mRNA (our work) and PAS-HDE region of mouse mH2A (sequence from Sun et al., Science, 2020), and their base pairing (bp) interactions with human U7 snRNA; structural details of colored bases are the same as in panels C and D. (**C**) Zoomed-in view of the pre-mRNA H2AC18 – U7 snRNA duplex region, showing the two additional base pairings (involving U40 and A41, indicated in red) predicted for the human histone mRNA. See also Figure 4C for more details about the whole duplex region. (**D**) Detailed view of the mouse mH2A – human U7 snRNA duplex structure (PDB accession number: 6V4X) (Sun et al., Science, 2020); for comparison, the same U7 region as in panel C is shown.

A

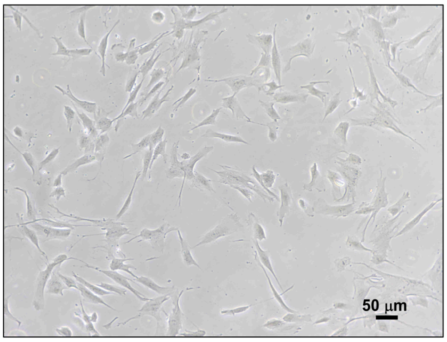

B

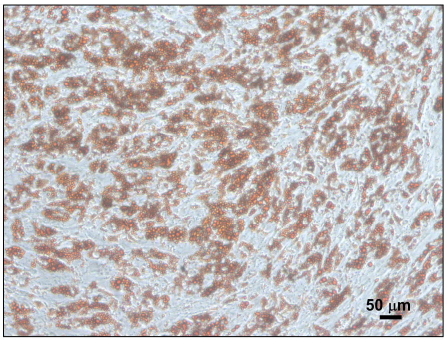

C

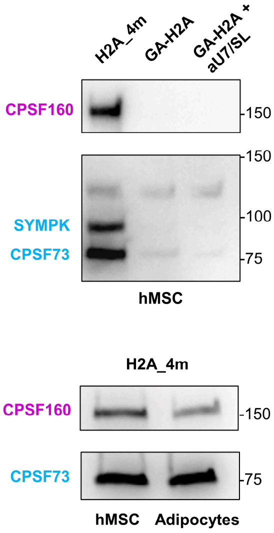

D

Uniprot	Protein (*Homo sapiens*)	Gene name	MW (Da)	hMSC Pep	hMSC SC	Adipocytes Pep	Adipocytes SC
Q10570	Cleavage and polyadenylation specificity factor subunit 1	CPSF1	160884	32	37	51	72
C9JEV9	Cleavage and polyadenylation specificity factor subunit 4	CPSF4	23653	3	3	6	6
O95639	Cleavage and polyadenylation specificity factor subunit 4	CPSF4	30255	3	3	5	5
Q16630	Cleavage and polyadenylation specificity factor subunit 6	CPSF6	59210	7	10	8	11
Q8N684	Cleavage and polyadenylation specificity factor subunit 7	CPSF7	52050	5	5	5	7
Q05048	Cleavage stimulation factor subunit 1	CSTF1	48358	13	16	14	20
P33240	Cleavage stimulation factor subunit 2	CSTF2	60959	2	2	5	5
Q9H0L4	Cleavage stimulation factor subunit 2 tau variant	CSTF2T	64437	2	2	6	6
Q12996	Cleavage stimulation factor subunit 3	CSTF3	82922	12	15	28	36
Q6UN15	Pre-mRNA 3'-end-processing factor FIP1	FIP1L1	66526	1	1	3	3
O43809	Cleavage and polyadenylation specificity factor subunit 5	NUDT21	26227	9	12	8	11
P62136	Serine/threonine-protein phosphatase PP1-alpha subunit	PPP1CA	37512	2	2	2	2
P62140	Serine/threonine-protein phosphatase PP1-beta subunit	PPP1CB	37187	1	1	3	3
P36873	Serine/threonine-protein phosphatase PP1-gamma subunit	PPP1CC	36984	2	2	2	2
Q9C0J8	pre-mRNA 3' end processing protein WDR33	WDR33	145891	3	3	9	14
Q9P2I0	Cleavage and polyadenylation specificity factor subunit 2	CPSF2	88487	15	19	27	36
Q9UKF6	Cleavage and polyadenylation specificity factor subunit 3	CPSF3	77486	14	18	22	34
Q92797	Symplekin	SYMPK	141148	5	6	17	22
Q8IV48	3'-5' exoribonuclease 1	ERI1	40064	8	8	3	3
P14678	Small nuclear ribonucleoproteins B and B'	SNRPB	24610	8	11	8	11
P62318	Small nuclear ribonucleoprotein Sm D3	SNRPD3	13916	11	14	8	15
P62304	Small nuclear ribonucleoprotein E	SNRPE	10804	2	2	2	2
P62306	Small nuclear ribonucleoprotein F	SNRPF	9725	4	4	4	5

E

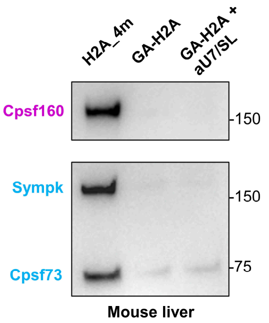

F

Uniprot	Protein (*Mus musculus*)	Gene name	MW (Da)	H2A_4m Pep	H2A_4m SC	H2A_4m iBAQ	GA-H2A Pep	GA-H2A SC	GA-H2A iBAQ
Q9EPU4	Cleavage and polyadenylation specificity factor subunit 1	Cpsf1	160818	7	7	1.31E+04			
Q99LC2	Cleavage stimulation factor subunit 1	Cstf1	48382	5	5	2.14E+04			
Q8BIQ5	Cleavage stimulation factor subunit 2	Cstf2	61341	7	10	1.66E+05			
Q8C7E9	Cleavage stimulation factor subunit 2 tau variant	Cstf2t	65862	1	1	4.56E+03			
Q99LI7	Cleavage stimulation factor subunit 3	Cstf3	82877	5	5	1.88E+04	N.D.		-
Q9D824	Pre-mRNA 3'-end-processing factor FIP1	Fip1l1	64959	3	3	1.40E+04			
Q9CQF3	Cleavage and polyadenylation specificity factor subunit 5	Nudt21	26240	1	1	2.77E+03			
O35218	Cleavage and polyadenylation specificity factor subunit 2	Cpsf2	88383	4	6	1.11E+04			
Q9QXK7	Cleavage and polyadenylation specificity factor subunit 3	Cpsf3	77505	3	4	2.13E+04			
P62307	Small nuclear ribonucleoprotein F	Snrpf	9725	2	2	2.58E+04			
P62320	Small nuclear ribonucleoprotein Sm D3	Snrpd3	13916	1	1	1.41E+04	1	1	6.56E+03

Figure S5 – H2AC18 assembles the endogenous canonical 3' end processing complex from human non-differentiated mesenchymal stem cells, terminally differentiated adipocytes and from mouse liver. Microscopy images of hMSC (**A**) and adipocytes (**B**) after staining procedure with Oil Red. (**C**) WB analysis of proteins belonging to the human endonuclease (CPSF73/CPSF3, SYMPK) and hPSF (CPSF160/CPSF1) modules, purified with H2A_4m (100 nM), GA-H2A (100 nM) and GA-H2A + aU7/SL (30 ug/ml each) from hMSC nuclear extracts (upper subpanel), or purified with H2A_4m (100 mM) from both hMSC and adipocytes (lower subpanel). (**D**) MS identification of human pre-mRNA processing factors, from hMSC and hMSC-derived adipocytes, associated to H2A_4m (1 µM RNA was used for this experiment) and belonging either to the canonical processing complex (purple) or histone processing complex (green) or to the shared cleavage factor (blue). The analysis was performed in triplicate, and the number of peptides and the spectral counts reported in the figure are representative values coming from one of the three replicates. (**E**) WB analysis of proteins belonging to the mouse endonuclease (Cpsf73/Cpsf3, Sympk) and mPSF (Cpsf160/Cpsf1) modules, purified with H2A_4m (100 nM), GA-H2A (100 nM) and GA-H2A + aU7/SL (30 ug/ml each) from nuclear extracts prepared from mouse liver tissues. (**F**) MS spectrometric identification of mouse pre-mRNA processing factors associated to H2A_4m and GA-H2A (100 nM of the two RNAs were used for this experiment). The same color code used in panel D applies to this panel. The reported values come from the analysis of a single biological replicate. Abbreviations: Pep – number of peptides identified; SC – spectral counts; iBAQ - Intensity-based absolute quantification.

Name	Structure	Sequence	Designation
wt H2AC18	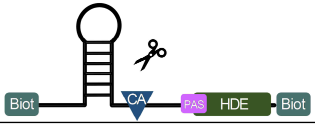	Biotin/ACUCAAAAGGCUCUUUUCAGAGCCACCCACGUUUUCAAAUAAAAGAGUUGUUAAUGCUGG/Biotin	From human histone H2AC18 3' UTR, substrate for activity assay, cleaved by 3' histone mRNA processing complex (this work)
H2A_4m	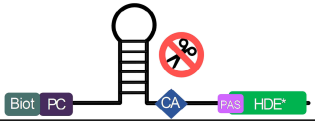	PCBiotin/18Sp/18Sp/ACUCAAAAGGCUCUUUUCAGAGCCACmCmCmAmCmGUUUUCAAAUAAAAGAGCUGUUACACCUGG	Modified from human histone H2AC18 3' UTR, bait for 3' complex purification and UV elution (this work)
38G/40A-H2A_4m (GA-H2A)	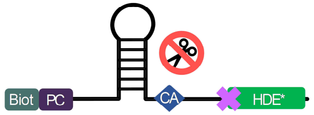	PCBiotin/18Sp/18Sp/ACUCAAAAGGCUCUUUUCAGAGCCACmCmCmAmCmGUUUUCAGAAAAAAGAGCUGUUACACCUGG	Modified from human histone H2AC18 3' UTR, bait for 3' complex purification and UV elution (this work)
H4_1m	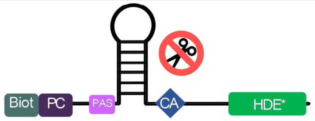	PCBiotin/18Sp/18Sp/CAAUAAAAGGCCCUUUUCAGGGCCACmCmCmUmAmCmUUUCUCAGCUGAAGAGCGGUAACACUGAG	Modified from human histone H4C11 3' UTR, bait for 3' complex purification and UV elution (this work)
2G/4A-H4_1m (GA-H4)	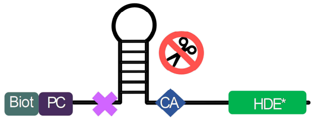	PCBiotin/18Sp/18Sp/CGAAAAAAGGCCCUUUUCAGGGCCACmCmCmUmAmCmUUUCUCAGCUGAAGAGCGGUAACACUGAG	Modified from human histone H4C11 3' UTR, bait for 3' complex purification and UV elution (this work)
mH2a*	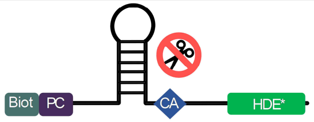	PCBiotin/18Sp/18Sp/CCAAAGGCUCUUUUCAGAGCCACmCmCmAmCmUGAAUCAGAUAAAGAGCUGUAACAC	Modified from mouse H2A 3' UTR, bait for 3' complex purification and UV elution (RNA seq. from Sun et al., Science, 2020)
Stem Loop (SL)	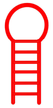	CCAAAGGCUCUUUUCAGAGCCACCCA	Selectively blocks endogenous SLBP (this work)
Antisense U7 (aU7)	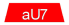	mAmAmGmAmGmCmUmGmUmAmAmCmAmCmU	Selectively blocks endogenous U7 snRNA and histone mRNA complex assembly (this work)
U7 snRNA	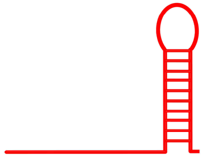	AGUGUUACAGCUCUUUUAGAAUUUGUCUAGUAGGCUUUCUGGCUUUUUACCGGAAAGCCC	Human sequence, forms a duplex with biotinylated RNA baits and blocks 3' pre-mRNA processing complex assembly
PAS	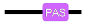	AACCUCCAAUAAACAAC	Blocks endogenous hPSF and canonical mRNA complex assebmly (RNA seq. from Sun et al., PNAS, 2018)
SV40-PAS	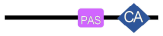	UUGUAACCAUUAUAAGCUGCAAUAAACAAGUUAmAmCmAmAmCAA	Blocks endogenous hPSF and canonical mRNA complex assebmly (this work)

Table S1 - List of RNA oligonucleotides used in this study, and their application. Stem loop sequences are underlined, both sequence and structural motifs are represented in the same color.

The following Tables are in separated .xlsx files containing raw data.

Table S2 – Mass spectrometry-based proteomic characterization of endogenous pre-mRNA 3'end processing complexes from HEK cells.

Table S3 – Mass spectrometry-based proteomic characterization of endogenous pre-mRNA 3'end processing complexes from human mesenchymal stem cells and adipocytes.

Table S4 – Mass spectrometry-based proteomic characterization of endogenous pre-mRNA 3'end processing complexes from mouse liver.
